# The harmful intestinal microbial community accumulates during DKD exacerbation and microbiome–metabolome combined validation in a mouse model

**DOI:** 10.3389/fendo.2022.964389

**Published:** 2022-12-19

**Authors:** Jin Shang, Wen Cui, Ruixue Guo, Yiding Zhang, Peipei Wang, Wei Yu, Xuejun Zheng, Ting Wang, Yijun Dong, Jing Zhao, Suying Ding, Jing Xiao, Zhigang Ren, Zhanzheng Zhao

**Affiliations:** ^1^ Department of Nephrology, The First Affiliated Hospital of Zhengzhou University, Zhengzhou, China; ^2^ Zhengzhou University, Zhengzhou, China; ^3^ Laboratory Animal Platform of Academy of Medical Sciences, Zhengzhou University, Zhengzhou, China; ^4^ Laboratory of Nephrology, The First Affiliated Hospital of Zhengzhou University, Zhengzhou, China; ^5^ Health Management Center, The First Affiliated Hospital of Zhengzhou University, Zhengzhou, China; ^6^ Department of Infectious Diseases, The First Affiliated Hospital of Zhengzhou University, Zhengzhou, China

**Keywords:** diabetic nephropathy, gut microbiota, untargeted (global) metabolomics, pathogenetic analysis, molecular mechanism

## Abstract

**Objective:**

Diabetic kidney disease (DKD) is one of the most prevalent complications of diabetes mellitus (DM) and is associated with gut microbial dysbiosis. We aim to build a diagnostic model to aid clinical practice and uncover a crucial harmful microbial community that contributes to DKD pathogenesis and exacerbation.

**Design:**

A total of 528 fecal samples from 180 DKD patients and 348 non-DKD populations (138 DM and 210 healthy volunteers) from the First Affiliated Hospital of Zhengzhou University were recruited and randomly divided into a discovery phase and a validation phase. The gut microbial composition was compared using 16S rRNA sequencing. Then, the 180 DKD patients were stratified into four groups based on clinical stages and underwent gut microbiota analysis. We established DKD mouse models and a healthy fecal microbiota transplantation (FMT) model to validate the effects of gut microbiota on DKD and select the potential harmful microbial community. Untargeted metabolome–microbiome combined analysis of mouse models helps decipher the pathogenetic mechanism from a metabolic perspective.

**Results:**

The diversity of the gut microbiome was significantly decreased in DKD patients when compared with that of the non-DKD population and was increased in the patients with more advanced DKD stages. The DKD severity in mice was relieved after healthy gut microbiota reconstruction. The common harmful microbial community was accumulated in the subjects with more severe DKD phenotypes (i.e., DKD and DKD5 patients and DKD mice). The harmful microbial community was positively associated with the serum injurious metabolites (e.g., cholic acid and hippuric acid).

**Conclusion:**

The fecal microbial community was altered markedly in DKD. Combining the fecal analysis of both human and animal models selected the accumulated harmful pathogens. Partially recovering healthy gut microbiota can relieve DKD phenotypes *via* influencing pathogens’ effect on DKD mice’s metabolism.

## Background

Diabetic kidney disease (DKD), one of the most prevalent complications of diabetes mellitus (DM) (1), is now the leading cause of end-stage renal disease (ESRD) worldwide (2). The outcomes of DKD is devastating and its diagnosis criteria mainly rely on clinical features and renohistopathological characteristics, in which it has been reported that only approximately 25% of clinically diagnosed DKD could be confirmed by renal biopsy (3, 4). Therefore, more advanced exploration is urgently needed to update the understanding of DKD pathogenesis to aid the real-life strategy of diagnosis and treatment.

Mounting evidence suggests that gut microbiota is now considered to be linked to many complicated diseases (5). The microorganic community varies substantially between healthy and sick conditions. It is generally believed that gut microbiota is associated with indigestible carbohydrate degradation (6), host immune tolerance promotion (7), and certain diseases’ development (8). In chronic kidney disease (CKD) and ESRD, gut microbiome interacts with impaired renal function through the gut–kidney axis (9). Gut microbiota also plays an important role in DM patients. Gut microbiota composition was altered significantly in DM patients (10), and distinct microbial profiles were found in DM patients with various serum uric acid levels (11). It has been widely reported that gut microbiota was involved in insulin resistance and glucose metabolism-related disorders (12, 13). In addition, diet adjustment targeting intestinal bacteria has great effects on improving hyperglycemia (14).

Herein, we characterized compositional changes of gut microbiota in 180 DKD and 348 non-DKD populations (138 DM patients and 210 healthy controls). Based on microbial comparison, we further constructed a gut microbiota-based classifier for DKD and non-DKDs in the discovery phase and validation phase, aiming to develop a novel diagnostic tool in clinical practice. Additionally, we stratified the DKD patients based on the clinical stages and compared the difference of intestinal microbial community among various DKD stages. DKD mouse models were constructed and underwent microbiota–metabolome combined analysis. After comprehensive delineation of intestinal microbial features in both human and animal levels, we selected potential harmful microbes and constructed their correlation relationship with renal clinical indices and important differential metabolites, aiming to uncover the microbial community that plays a role in DKD exacerbation and its underlying pathogenetic mechanism.

## Materials and methods

### Participant enrollment

The study was designed based on the principles of prospective specimen collection and retrospective blinded evaluation (15). The patients diagnosed as DKD or DM and hospitalized in the First Affiliated Hospital of Zhengzhou University from October 2018 to October 2019 were enrolled. Fecal samples of healthy controls were obtained from the physical examination center. Our study followed the principles of the Declaration of Helsinki. The Ethics Review Committee approved all experimental processes (2019-KY-361). All individuals knew their rights and signed written informed consents before sample collection.

Diagnostic criteria for DKD were at least 5 years history of diabetes complicated with repeated albuminuria (urinary protein/creatinine ≥ 30 mg/g) or macro-protein urine (16, 17). The exclusion criteria of the study are as follows: (1) patients combined with other secondary kidney diseases (e.g., infection, lupus, vasculitis, hepatitis B, and other secondary kidney diseases); (2) pure primary glomerulonephritis confirmed by renal biopsy; (3) the patients whose DKD was controlled when the sample was collected and the urine protein turned negative; (4) application of antibiotics or probiotics within 3 months prior to sample collection; and (5) incomplete information. Healthy volunteers were screened in terms of the following inclusion criteria: (1) normal urine test, serum albumin, and creatinine (Cr); and (2) normal blood glucose and glycosylated hemoglobin (GHb). The volunteers who reported basic diseases or with antibiotics application before sample collection were not included. Finally, we consecutively recruited 180 DKDs, 138 DMs, and 210 healthy controls.

### 16S rRNA sequencing

A total of 528 fecal samples of participants were collected and subjected to 16S rRNA gene sequencing. Fecal samples collected from all participants were temporarily stored in a 4°C environment and then transferred to a −80°C environment within 2 h for further analysis. DNA extraction from fecal samples was performed as previously described (18, 19) using E.Z.N.A.^®^ Stool DNA Kit (Omega Bio-tek, Inc., GA). The detailed information of DNA extraction were described in **Supplemental Document 1**. Shanghai MoBio Biomedical Technology Co. Ltd. provided technical support using the Miseq platform (Illumina Inc., USA) per the manufacturer’s protocols. The primers F1 and R2 (5’-CCTACGGGNGGCWGCAG-3’ and 5’-GACTACHVGGGTATCTAATCC-3’), which correspond to positions 341 to 805 in the *Escherichia coli* 16S rRNA gene, were used to amplify the V3–V4 region by PCR. PCR amplification of the V3–V4 region of the 16S rRNA gene and Illumina paired-end sequencing was performed according to a previous description. To obtain the clean data, we treated the raw data using USEARCH (version 11.0.667) with the following criteria: (1) sequences of each sample were extracted using each index with zero mismatch; (2) sequences with overlap less than 16 bp were discarded; (3) sequences less than 400 bp after merge were discarded; (4) sequences with the error rate of the overlap greater than 0.1 was discarded. The quality-filtered sequences were clustered into unique sequences and sorted in order of decreasing abundance. According to the UPARSE OTU analysis pipeline, the representative sequences were identified using UPARSE, and singletons were omitted in this step. Operational taxonomic units (OTUs) were obtained based on 97% similarity after chimeric sequences removed using UPARSE (version 7.1 http://drive5.com/uparse/), and were annotated using the SILVA reference database (SSU138) (Edgar 2013). The phylogenetic affiliation of the 16S rRNA gene sequence was analyzed with a confidence threshold of 70% (20).

### OTU clustering and gut microbe-based ROC construction

Chimeric sequences were removed using UPARSE (version 7.1 http://drive5.com/uparse/). Then, we classified OTUs with 97% similarity (21). To analyze the phylogenetic affiliation of 16S rRNA gene sequence, we used RDP Classifier (http://rdp.cme.msu.edu/) against the Silva (SSU123) 16S rRNA database with a confidence threshold of 70%. Gut microbiota composition and functional changes were compared among different groups. Detailed statistical bioinformatic analysis and tests were described in **Supplementary File 2**.

Using the abundance profiles of OTU, fivefold cross-validation was implemented on a random forest model to characterize important OTUs (importance value > 0.001) for classification of 120 DKDs and 232 non-DKDs (92 DMs and 140 Con) and calculate the possibility of disease (POD). Then, we performed receiver operating characteristic (ROC) curve construction for the discovery cohort and validation cohort (22). Area under ROC curve (AUC) values were generated in R (http://www.R-project.org/).

### Animal modeling and experiment design

All animal experiments were approved by Ethical Committee of Experimental Animal Care of First Affiliated Hospital of Zhengzhou University (2021-KY-0162). A total of 30 6-week-old male C57/BL6 mice were purchased from the Animal Center of Zhengzhou University. Fecal microbiota transplantation (FMT) and antibiotic cocktails gavage were performed on mice to demonstrate the relationship between the intestinal microbiota and DKD phenotypes. Broad-spectrum antibiotics were used to clear gut microbiota as previously described (23, 24). Intraperitoneal streptozotocin (STZ) injection accompanied with high-fat diet (HFD) feeding (45% fat, XTHF45-1, Jiangsu, China) was used to induce DKD. The control group was fed with chow diet (SWS9102, Jiangsu, China). The fecal samples of the mouse model were collected and subjected to 16S rRNA sequencing. Detailed information of the modeling process, fecal bacteria solution preparation, antibiotic formula, and sample collection is shown in **Supplementary File 3**.

The mice were randomly divided into four groups: (1) the healthy control (Con) group (*n* = 7); (2) the canagliflozin-treated (10 mg/kg/day) DKD group (Cana group) as positive control (*n* = 8) (25); (3) the DKD group (*n* = 8); and (4) the broad-spectrum antibiotic cocktail-clearing DKD mice transplanted with fecal microbiota of healthy mice (FMT) group (*n* = 7). Detailed information of the experiment design and sample collection plan is shown in **Supplementary File 3**.

### Clinical and pathological evaluation of mouse models

Random blood glucose level was measured once a week. Total urinary protein/urinary creatinine (T/Cr) was used to evaluate the severity of DKD and presented as scatter charts. Hematoxylin–eosin (HE) staining was used to observe glomerular histomorphology. Periodic acid–Schiff (PAS) and Masson staining were used to observe fibrosis and carbohydrate deposition in kidney tissue, respectively. Detailed staining processes are presented in **Supplementary File 4**. Transmission electron microscopy (TEM) was used to visualize and assess the submicroscopic pathological changes and glomerular immune complex deposition in DKD.

### Metabolic profile delineation of mouse models

The 17 serum samples (6 DKDs, 6 FMTs, and 5 HCs) of mice were subjected to ultra-high-performance liquid chromatography-mass spectrometry (UPLC-MS)-based untargeted metabolomic analysis to globally describe the serum metabolic features of DKD mice and the difference among DKD, Con, and FMT groups. All detected metabolites were identified by MS and MS/MS fragment through Progenesis QI (WaterCorporation, Milford, USA) with several mainstream public databases (http://www.hmdb.ca/, https://metlin.scripps.edu/). Principal component analysis (PCA) and Orthogonal Partial Least-Squares Discrimination Analysis (OPLS-DA) were performed to identify the discrimination of serum metabolites. Based on OPLS-DA analysis, the metabolites with variable importance in projection (VIP) > 1 are recognized as important variables. VIP represents the ability to extract variables of differentiation among groups. Important differential metabolites were defined as those with VIP > 1.0 obtained from OPLS-DA and adjusted *p*-values < 0.05. Detailed information of chemicals and equipment, sample processing, UPLC-MS analysis, and bioinformatic and statistical analysis are shown in **Supplementary File 5**.

## Results

### Baseline characteristics of participants

**Figure 1:**
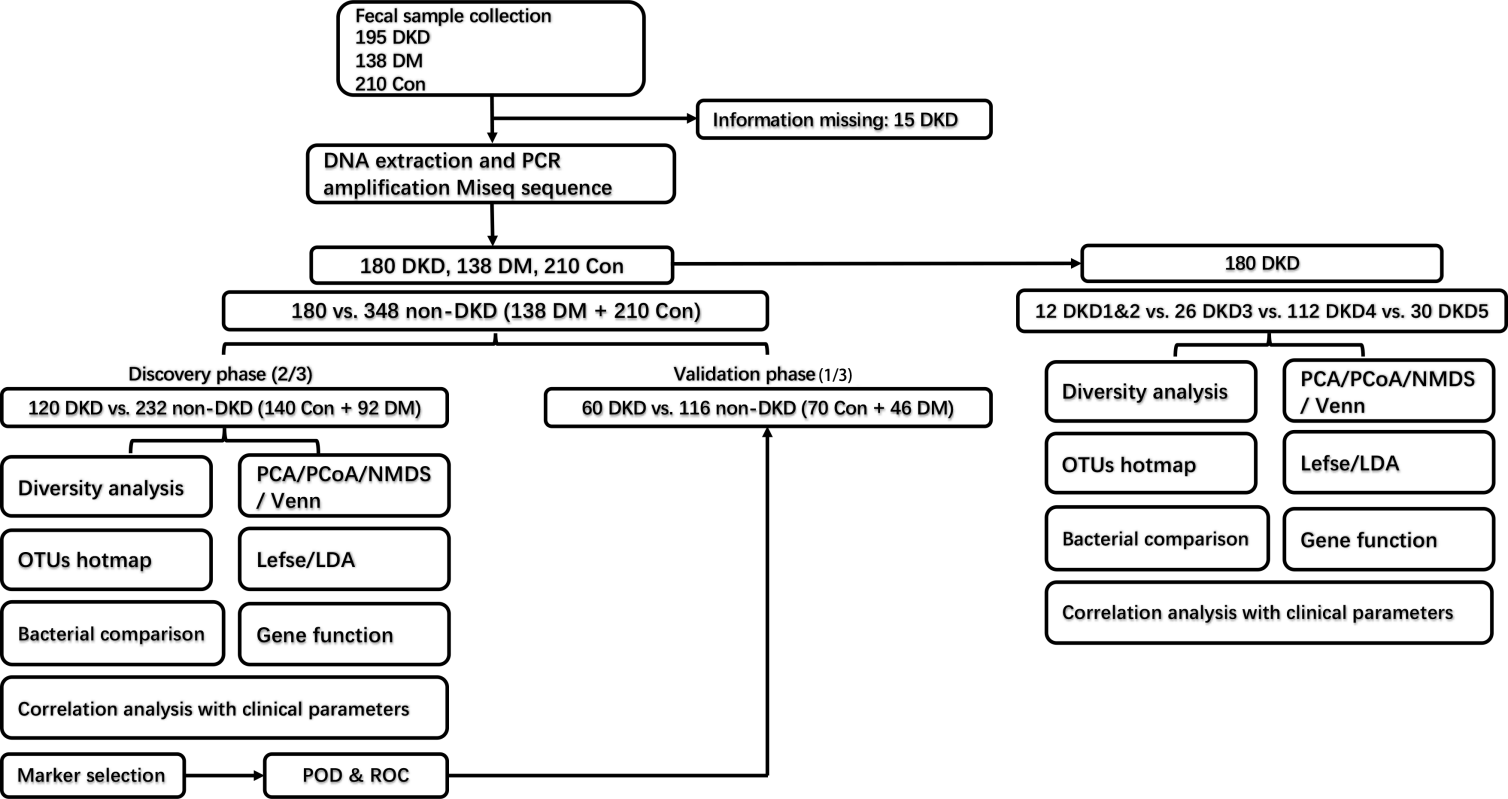
Experimental flowchart. DKD, diabetic kidney disease; DM, diabetes mellitus; Con, healthy controls; OTU, operational taxonomy units.

The grouping and study process are shown in **Figure 1**. As shown in **Table 1**, when compared with the DM and healthy control (DMHC) group, DKD patients in the discovery group showed significantly decreased estimated glomerular filtration rate (eGFR) and serum albumin (Alb) level, while increased 24-protein and serum creatinine. No antibiotic treatment was given in all patients before sample collection. There is no significant difference in body mass index (BMI) between DKD (*n* = 180) and DM (*n* = 138) patients, which is 24.82 (23.91, 25.38) kg/m^2^ *vs*. 24.79 (22.51, 26.24) kg/m^2^ (*p* value > 0.05), respectively.

**Table 1:** Demographic characteristics of participants in discovery and validation cohorts.

Clinical indices	Discovery cohort			Validation cohort		
	DKD(n=120)	DMHC(n=232)	P-Value	DKD(n=60)	DMHC(n=116)	P-Value
Gender			0.063			0.172
Male	75	121		37	59	
Female	45	111		23	57	
Age	56(49, 62)	51(46, 57)	<0.001	55±12	52±10	0.077
DM course(month)	114(36, 180)	0(0, 23)	<0.001	90(8, 156)	0(0, 8)	<0.001
eGFR(mL/min)	50.11(25.19, 94.86)	101.94(93.01, 107.97)	<0.001	61.86(25.81, 97.75)	101.11(94.96, 107.78)	<0.001
SBP(mmHg)	139.4±18.3	122.7±15.1	<0.001	135(124, 150)	122(113, 129)	<0.001
DBP(mmHg)	80.7±10.1	75.8±9.7	<0.001	82±13	75±9	0.001
Ghb(%)	7.39(6.60, 8.74)	5.57(4.87, 5.94)	<0.001	7.59(6.68, 8.93)	5.60(5.02, 6.00)	<0.001
24h-pro(g)	2.94(0.60, 6.01)	0.06(0.06, 0.13)	<0.001	1.93(0.75, 5.03)	0(0, 0.02)	<0.001
Cr(μmol/L)	120.5(74.0, 237.7)	65.0(55.0, 76.0)	<0.001	101.5(71.0, 218.4)	62.0(55.5, 75.3)	<0.001
Alb(g/L)	35.7(28.3, 40.7)	45.9(42.0, 48.1)	<0.001	34.9±7.3	43.3±10.2	<0.001
	DKD(n=180)			DM(n=138)		
BMI(kg/m^2^)	24.82(23.91-25.38)			24.79(22.51-26.24)		0.115

Normal distribution was measured by K-S test. Subsequent analysis between groups were completed by LSD t-test. Variances between DN and MN were analyzed by t-test. χ^2^ test was used to compare categorical variables. DM course, course of diabetes mellitus; eGFR, estimated glomerular filtration rate; SBP, systolic blood pressure; DBP, diastolic blood pressure; Ghb, glycosylated hemoglobin; 24h-pro, 24-h urine protein; Cr, creatinine; Alb, serum albumin. BMI, body mass index.

### Gut microbiome profiles were altered dramatically in DKD patients

**Figure 2:**
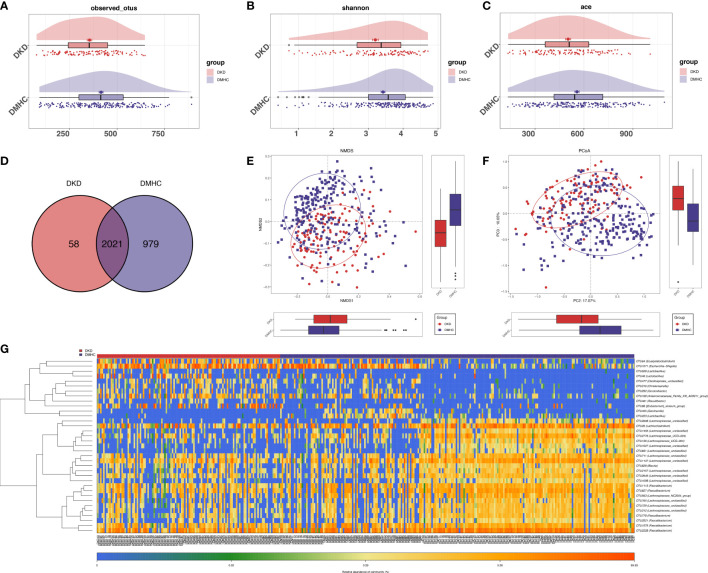
Bacterial diversity in discovery cohort (DKD = 120, DM = 140, and Con = 92). α diversity: Bacterial richness and diversity of DKD and non-DKD group comparison were assessed by observed OTUs **(A)** and Shannon/Ace indices **(B, C)**, respectively. Venn diagram **(D)** showed that observed OTUs among two groups. β diversity: NMDS analysis **(E)** based on unweighted UniFrac distance (ANOSIM, *R* ^2^ = 0.1123, *p* = 0.0001). PCoA analysis **(F)** was measured by unweighted UniFrac distance at the OUT level. Adonis revealed that unweighted analysis taking OTU abundance into account could better reflect the spatial differences among two groups (*R* ^2^ = 0.0247, *p* = 0.001). **(G)** Distribution of key OTUs between DKDs and non-DKDs. Through Wilcoxon rank-sum test, a total of 35 OTUs with *p*-value > 0.05 and abundance > 0.03% were considered as key lineages for DKD. Blue color represented lower abundance. Orange color represented higher abundance. PCoA, principal coordinate analysis; PC, principal component, PC1, PC2, and PC3. NMDS, non-metric multidimensional scaling analysis; Adonis, permutational/nonparametric multivariate analysis of variance; ANOSIM, analysis of similarities.

The estimated OTU diversity (including richness and evenness) in DKD was significantly decreased when compared with that of DMHC (**Figures 2A–C**, **S1**, **S2**, **Table S1**). As exhibited by overlaps in the Venn diagram, 2,021 of the total 3,058 OTUs were shared by both groups and 58 OTUs were specific for the DKD group (**Figure 2D**, **Table S2**). Beta-diversity showed separating distribution of bacterial community between DKD and DMHC groups (Adonis for PCoA, *R* ^2^ = 0.025, *p* = 0.001, **Figure 2E**; ANOSIM for NMDS, *R* ^2^ = 0.112, *p* = 0.001, **Figures 2F**, **S2**, **Table S3**). Gut microbes with mean abundance larger than 0.003% and *p*-value lower than 0.05 through Wilcoxon test were considered as key OTUs. Thirty-five OTUs were selected and presented as heatmap showing an apparently separated distribution between DKD and DMHC groups (**Figure 2G** and **Table S4**).

**Figure 3:**
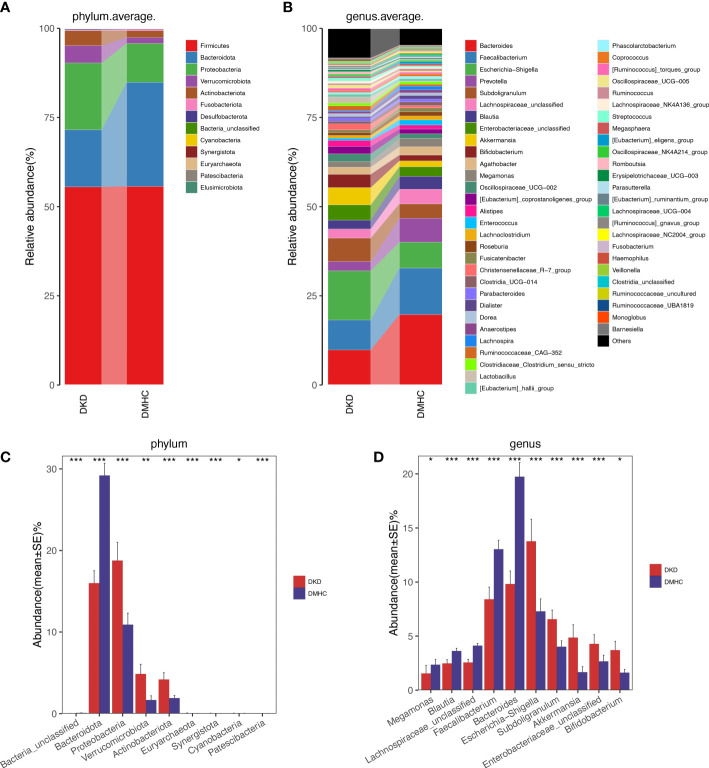
Microbial communities altered between DKD patients and non-DKD populations. In discovery phase, composition of gut microbiome at the phylum **(A)** and genus **(B)** level. Kruskal–Wallis rank-sum test was used to compare and identify significantly different bacteria at the phylum **(C)** or genus **(D)** level. Only bacteria with gradually increased or decreased abundance between two groups were shown. *p < 0.05, **p < 0.01, ***p < 0.001.

The average gut microbiome in DMHC and DKD groups was dominated by phyla *Firmicutes, Bacteroidetes, Proteobacteria*, and *Verrucomicrobia* (all accounting for more than 95% in both groups, **Figures 3A** and **S3**). At the genus level, bacterial frameworks of both groups composed of 103 genera were displayed in a bar plot (**Figure 3B**). Wilcoxon test showed that the abundance of phyla *Proteobacteria, Actinobacteriota, Synergistota, Euryarchaeota, Patescibacteria, Verrucomicrobiota*, and *Cyanobacteria* were accumulated in DKD when compared with those in the DMHC group (**Figure 3C** and **Table S5**), while the abundance of phyla *Bacteroidota* and *Bacteria_unclassified* was depleted in DKD (**Figure 3C** and **S3**, **Table S6**, **S7** and **S8**). At the genus level, we observed expansion of 107 genera in DKD. Of these discriminatory genera, *Escherichia-Shigella*, *Subdoligranulum, Enterobacteriaceae_unclassified, Akkermansia, Bifidobacterium, [Eubacterium]_siraeum_group, Negativibacillus*, and *Acetanaerobacterium* were more enriched in the DKD group than those in DMHC group, while *Bacteroides* and *Faecalibacterium* were more depleted (**Figure 3D**, **Table S9**).

### Identification and construction of microbial OUT-based diagnostic model

**Figure 4:**
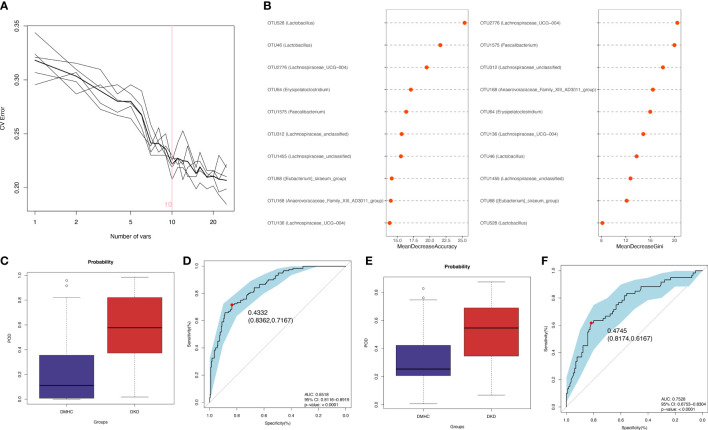
Diagnostic outcomes for DKD and non-DKD in discovery phase and validation phase. **(A, B)** Predictive performance of microbial combinations including different key OTU number (computed by 5-fold cross-validation and random forest). POD index in discovery phase **(C)** or validation phase **(E)** was used to estimate diagnostic efficiency for DKD. Integrating fivefold cross-validation with random forest model, ten microbial OTUs was identified as optimal diagnostic biomarkers. **(D)** ROC curve of the DKD-related classifier using 10 microbial biomarkers to distinguish 120 DKDs from 232 non-DKDs. **(F)** AUC in validation phase verified the predictive power of diagnostic model in 60 DKDs and 116 non-DKDs. Corresponding 95% CI and P value were shown in graph. CV, cross-validation; POD, possibility of disease; ROC, receiving operational curve; AUC, area under the curve; CI, confidence intervals.

The LEfSe algorithm was performed and 38 genera with LDA score > 3.0 and *p*-value < 0.05 were identified as a significantly different gut microbiome (**Figure S4B** and **Table S10**). With five trials of fivefold cross-validation performed on the random forest model, 10 optimal OTUs were set as identification biomarkers for 120 DKDs and 232 non-DKDs samples (92 DMs and 140 controls) in the discovery phase (**Table S11**) and underwent correlation analysis with clinical variables (**Figures 4A** and **S4C**, **Table S12**). In the discovery phase, a higher average POD value was displayed in the DKD group than in the DMHC group (*p* < 0.001, **Figure 4C** and **Table S13**). The POD index-based AUC of the training set was 85.18% (95% CI 81.16% to 89.19%, **Figure 4D**). In the validation cohort including 60 DKDs and 116 DMHCs, the average POD value in the DKD group was significantly higher than in DMHCs (*p* < 0.001, **Figure 4E** and **Table S14**) and the AUC was 75.28% (95% CI 67.53% to 83.04%, **Figure 4F**). These results suggested that microbiota-targeted markers could achieve high diagnostic efficiency for separating DKD patients from non-DKDs.

### DKD exacerbation induces a certain trend of microbial disturbance

**Figure 5:**
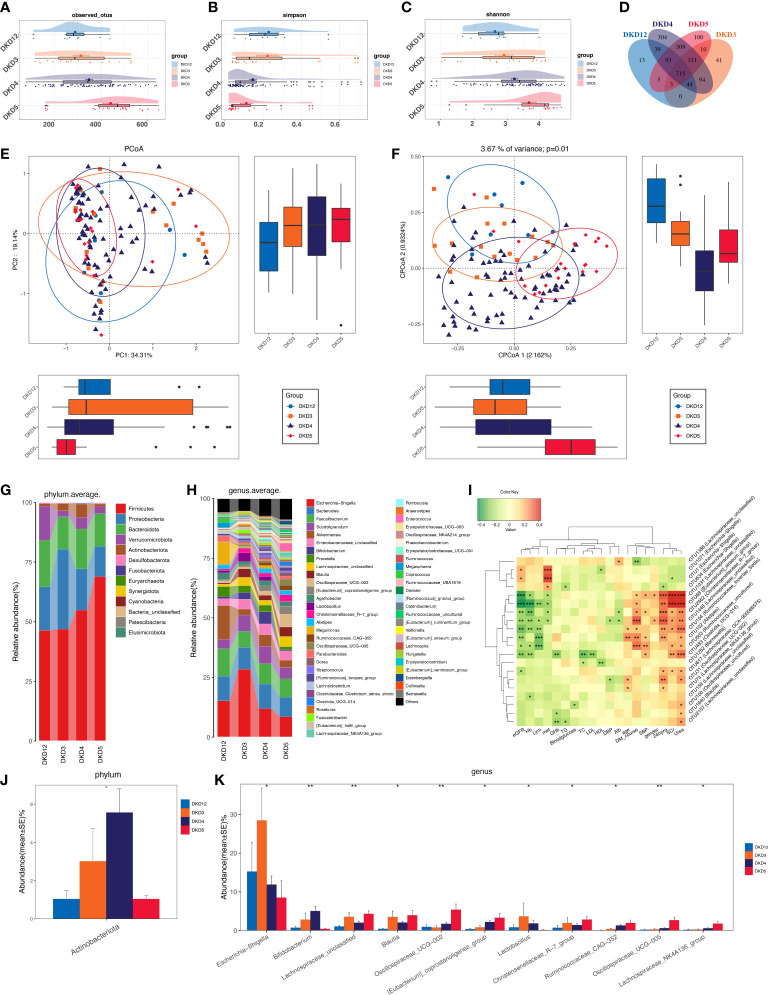
Grouping DKD patients (12 DKD 1&2, 26 DKD 3, 112 DKD 4, and 30 DKD 5) based on clinical stages and their analysis of intestinal bacterial diversity and microbial communities’ alteration during DKD exacerbation and Spearman’s correlation analysis between key OTUs and crucial clinical indices. The observed OTUs **(A)** and Simpson/Shannon indices **(B, C)** were used to assess α diversity. Venn diagram presented the observed OTUs among four groups **(D)**. PCoA analysis **(E)** was measured by Bray–Curtis distance at the I level. CAP analysis **(F)** was measured by unweighted UniFrac distance. Intestinal microbial composition at the phylum **(G)** and genus **(H)** level. Kruskal–Wallis rank-sum test was used to compare and identify significantly altered bacteria at the phylum **(J)** or genus **(K)** level. Only bacteria with gradually increased or decreased abundance between two groups were shown. Spearman’s correlation relationship between key altered OTUs and crucial clinical indices were presented as heatmap **(I)**. PCoA, principal coordinate analysis; PC, principal component, PC1, PC2, and PC3. *p < 0.05, **p < 0.01, ***p < 0.001.

As we revealed that gut microbiota of DKD patients was significantly changed when compared with that of the DMHC group, we then stratified DKD patients based on the different clinical stages and set four groups (DKD stage 1&2, stage 3, stage 4, and stage 5) to confirm the findings above and further delineate the changing trend of microbial community during DKD exacerbation. The estimated OTU diversity was dramatically increased during the DKD deterioration (**Figures 5A–C**, **Table S15**) and showed obviously separating distribution on PCoA and CAP analysis (Adonis for PCoA, *R* ^2^ = 0.037, *p* = 0.011, **Figure 5E**; ANOVA-like permutation for CAP, *p* = 0.004, **Figure 5F**, **Table S17**). The Venn diagram showed that 715 of the total 2,079 OTUs were shared by all four groups, and 13, 41, 304, and 100 OTUs were specific for DKD 1&2, 3, 4, and 5 groups (**Figure 5D**, **Table S16**), respectively. The average gut microbiome in all four DKD groups was dominated by phyla *Firmicutes, Bacteroidota, Proteobacteria*, and *Verrucomicrobia* (all accounting for more than 95% in both groups, **Figures 5G** and **S5**). Wilcoxon test showed that the abundance of phyla *Actinobacteriota* is the only one that was significantly different (*p* = 0.026) among the four groups and accumulated in DKD4 (**Figures 5J** and **S5**, **Tables S18**-**S21**). At the genus level, 44 genera were significantly different among four DKD groups (**Figure 5H** and **Table S22**). Notably, 31 of 44 genera were dramatically accumulated in the DKD 5 group compared to those in the other three groups. Surprisingly, 16 of these 31 genera were also the genera that significantly accumulated in the DKD patients when compared with those in the DMHC group (**Table 2**), which may be selected as candidate pathogens. Among them, *[Eubacterium]_siraeum_group, Negativibacillus*, and *Acetanaerobacterium* are the genera that were most significantly accumulated in the DKD 5 group (**Table 2**).

**Table 2:** The potentially harmful significantly accumulated genus shared by both DKD when compared with DMHC and DKD5 when compared with other DKD stages.

Genus		DKD V.S. DMHC					DKD5 V.S. Other stages			
	DKD mean abundance	DMHC mean abundance	P-Value	Significance mark	DKD1&2 mean abundance	DKD3 mean abundance	DKD4 mean abundance	DKD5 mean abundance	P-Value	Significance mark
Christensenellaceae_R-7_group	0.016780475	0.00634137	<0.001	***	0.00727363	0.01963563	0.01410525	0.02846495	0.01374242	*
Oscillospiraceae_UCG-005	0.009059783	0.00383319	<0.001	***	0.00229413	0.00328644	0.00633195	0.0267505	0.00208477	**
[Eubacterium]_siraeum_group	0.003143658	5.10E-04	<0.001	***	3.46E-05	9.68E-04	0.00333049	0.00541825	<0.001	***
Ruminococcaceae_Incertae_Sedis	0.001846742	7.17E-04	<0.001	***	7.87E-04	0.00108463	0.00136208	0.0047221	0.00860697	**
Eisenbergiella	0.00259155	5.45E-05	<0.001	***	7.21E-04	8.13E-05	1.08E-04	0.01478735	0.01056184	*
[Clostridium]_methylpentosum_group	2.30E-04	4.13E-05	<0.001	***	6.79E-05	3.89E-05	2.36E-04	4.25E-04	0.02048064	*
Candidatus_Soleaferrea	1.48E-04	3.63E-05	<0.001	***	4.13E-05	1.10E-04	1.07E-04	3.75E-04	0.02619775	*
Pygmaiobacter	1.20E-04	4.63E-06	<0.001	***	7.13E-06	8.88E-06	1.04E-04	3.12E-04	0.00382388	**
Anaerofustis	2.30E-05	3.68E-06	<0.001	***	3.29E-05	1.61E-05	1.32E-05	6.21E-05	0.025791	*
[Eubacterium]_coprostanoligenes_group	0.020859025	0.01300594	0.00369213	**	0.00383125	0.00837588	0.02203784	0.03317715	0.04111925	*
Ruminococcaceae_CAG-352	0.012251092	0.00535581	0.0022268	**	7.65E-04	0.00393394	0.01322849	0.0197852	0.0249018	*
Moryella	1.12E-04	6.25E-05	0.00481505	**	7.03E-05	1.19E-04	8.25E-05	2.34E-04	0.00809893	**
Anaerofilum	4.47E-05	1.05E-05	0.0027824	**	3.00E-06	4.81E-06	2.78E-05	1.58E-04	0.00643322	**
Acetanaerobacterium	1.71E-05	3.23E-06	0.00566189	**	0	2.44E-06	1.77E-05	3.36E-05	<0.001	***
Oscillospiraceae_UCG-002	0.021873833	0.01287019	0.02670477	*	0.00964975	0.00795313	0.01760568	0.054119	0.00702877	**
Negativibacillus	5.16E-04	3.48E-04	0.04795034	*	9.89E-04	5.95E-05	3.62E-04	0.0012797	<0.001	***

DKD, Diabetic Kidney Disease; DMHC, Diabetes Mellitus and Healthy control; *P<0.05, **P<0.01, ***P<0.001.

### Healthy FMT alleviates DKD severity efficiently

**Figure 6:**
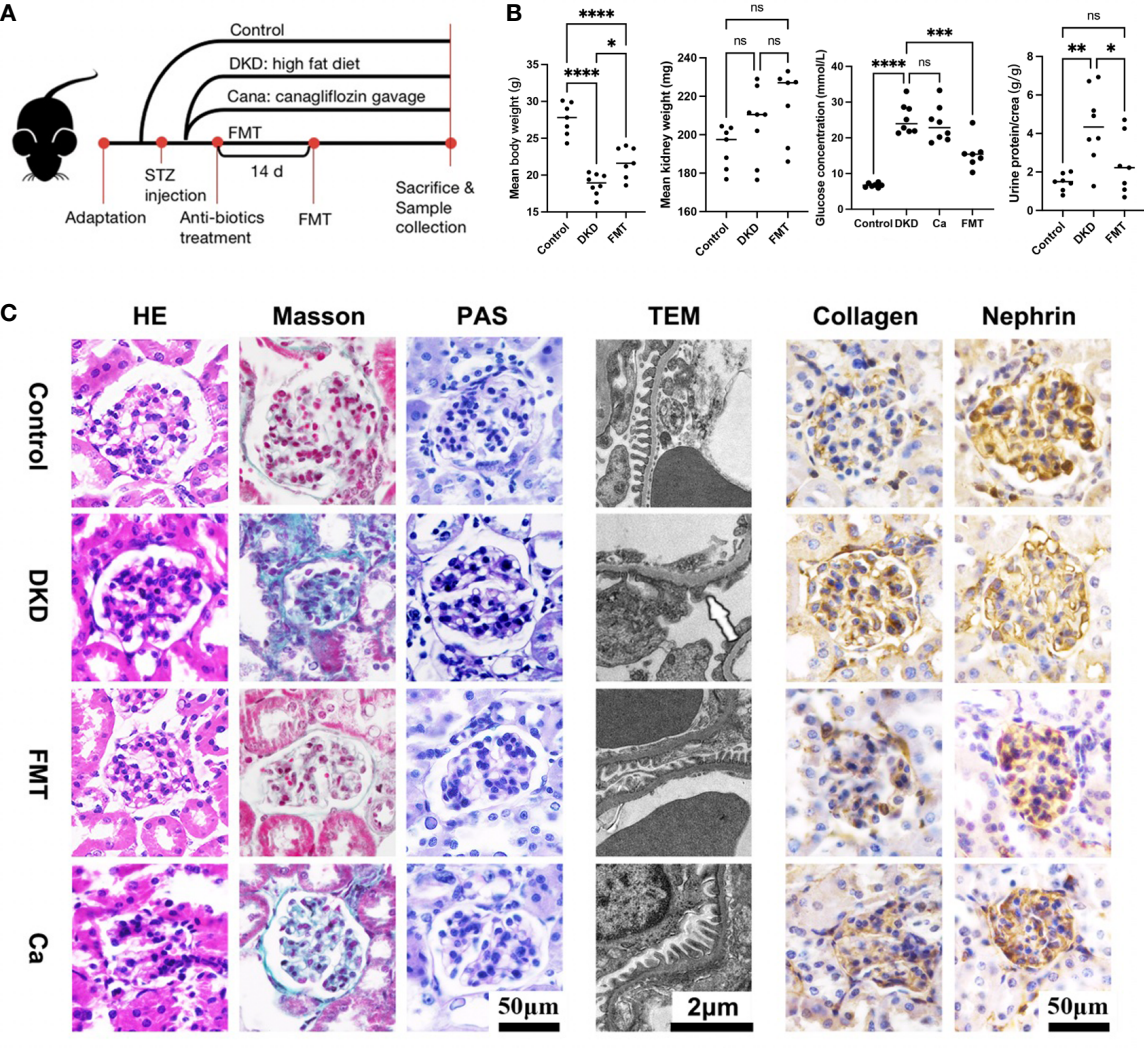
Clinical phenotyping and pathological features of mouse models. Flow chart of mouse experiment **(A)**. Scatter plots showed the body weight, mean kidney weight,serum glucose concentration and urine protein to creatinine ratio of each group at the end of the experiment **(B)**. HE, Masson and PAS staining of glomeruli in each group and EM showed detailed pathological changes in each group **(C)**. FMT, fecal microbiota transplantation; STZ, streptozotocin; Ca, Canagliflozin; HE, hematoxylin-eosin staining; PAS, periodic acid-schiff staining; EM, electron microscopy. *p<0.05; **p<0.01; ***p<0.001; ****p<0.0001. ns, no significance.

To uncover the underlying relationship between gut microbiota alterations and DKD severity, we constructed DKD mouse models and implemented animal experiments. The grouping and experimental design are illustrated in **Figure 6A**. The DKD mouse models were established successfully as shown by the fact that as the body weight of all DKD mouse models was significantly decreased, the mean kidney weight, blood glucose level, and urine T/Cr were significantly increased compared with the Con group (**Figure 6B**). After clearing the gut microbiota of DKD mouse models and recovering the normal intestinal flora by healthy FMT, the clinical features except mean kidney weight were significantly alleviated, shown as increased body weight, decreased blood glucose level, and urine T/Cr (**Figure 6B**). The DKD mice treated with canagliflozin, an efficient blood sugar-lowering agent that was widely used in DKD patients, were set as positive control. However, the blood sugar level of canagliflozin was not significantly different with that of the DKD group. Surprisingly, the blood sugar-lowering effect induced by FMT was stronger than the canagliflozin-treating group (**Figure 6B**) and FMT significantly decreased the blood glucose level than the DKD group (*p*-value < 0.0001), indicating the underlying protective role of the normal gut microbial community.

In terms of histopathological features, the successful DKD modeling was verified again based on the typical pathological lesions, like significantly enlarged and sclerotic glomeruli shown on H&E staining, and dramatic carbohydrate deposition and fibrosis on PAS and Masson staining, respectively (**Figure 6C**). Immunohistochemistry showed relatively enhanced expression of collagen and suppressed nephrin expression compared with the Con group, indicating more severe fibrosis and renal injury (**Figure 6C**). TEM also showed dramatic basement membrane thickening, diffuse mesangial hyperplasia, foot process effacement, and KW nodule formation (**Figure 6C**). After clearing the gut microbiota of DKD mice and reconstructing the normal intestinal flora, glomerular sclerosis and fibrosis, glomerular injury, basement membrane thickening, and mesangial hyperplasia were significantly alleviated (**Figure 6C**), which was consistent with the clinical feature changes above. It needs to be mentioned that, when compared with the DKD mice treated with canagliflozin, the pathological lesion-alleviating effects of FMT were more pronounced (**Figure 6C**).

### The gut microbiota of FMT mice were disparate with that of the DKD group

**Figure 7:**
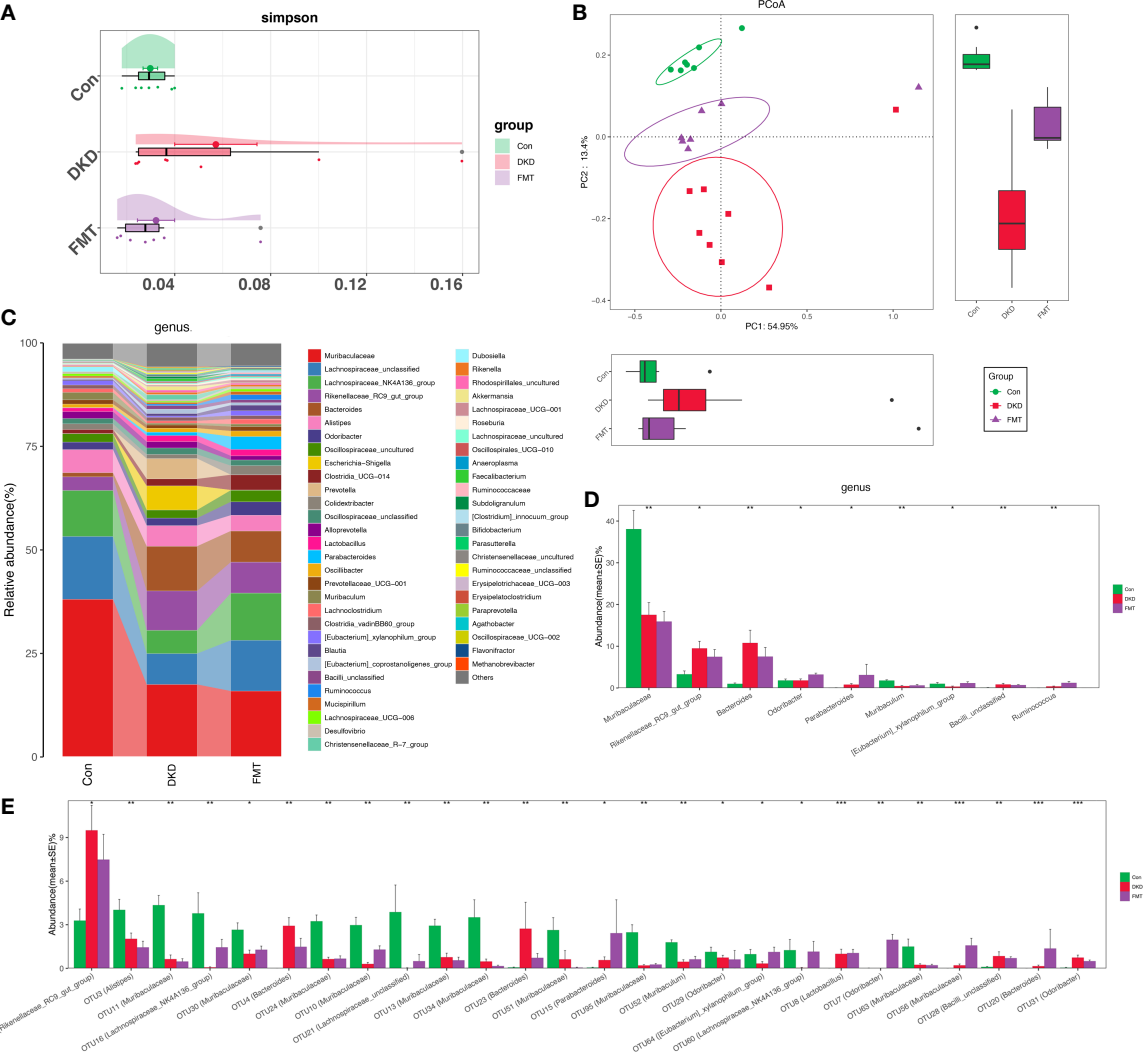
Bacterial diversity and compositional alteration analysis of mouse models. Simpson indices **(A)** and PCoA analysis **(B)** were used to assess α and β diversity, respectively. PCoA analysis was measured by unweighted UniFrac distance at OTU level. Adonis revealed that unweighted analysis taking OTU abundance into account could better reflect the spatial differences among three groups (*R* ^2^ = 0.2233, *p* = 0.0001). Intestinal microbial composition at the genus level **(C)**. Kruskal–Wallis rank-sum compares and identifies significantly altered bacteria at the genus and OTU level and the genus **(D)** or OTU **(E)** with gradually increased or decreased abundance between three groups was shown.

As we revealed that healthy FMT can dramatically alleviate DKD severity indicating that specific gut microbiota alterations may play a key role in DKD pathogenesis, we further aim to delineate such specific changes between healthy FMT mice and DKD mice. Bacterial diversity was significantly recovered after FMT when compared with the DKD group (**Figure 7A**). PCoA showed a distinct separating distribution of the bacterial community between the FMT and DKD group (Adonis for PCoA, *R* ^2^ = 0.2233, *p* = 0.0001, **Figure 7B**, **Table S23**). Average bacterial communities at the genus level among Con, FMT, and DKD groups were presented as a bar plot (**Figure 7C**). Kruskal–Wallis test at the genus level showed that genera *Odoribacter, Parabacteroides, Ruminococcus, Mycoplasma*, and *Enterobacteriaceae_unclassified* were relatively accumulated in the FMT group when compared with those in DKD and Con group (**Figure 7D**, **Table S24**). All findings above demonstrated a clear discrimination of microbial diversity or composition in the FMT group from the DKD group.

### The common microbial community was shared in both human and animals with more exacerbating DKD

Considering that the microbial community of the patients with more exacerbating DKD phenotypes (i.e., DKD vs. DMHC and DKD stage 5 *vs*. other DKD stages) shared the common microbial profiles at the genus level (**Table 2**), we further selected the common accumulated OTUs shared by both humans and mice with more exacerbating phenotypes (i.e., compared DKD *vs*. DMHC group, DKD5 *vs*. relatively relieved DKD stages and DKD mice *vs*. Control/FMT mice), aiming to localize the targeted harmful pathogens that may play a role in DKD exacerbation. There were 14 common OTUs relatively accumulated in three groups of subjects with more exacerbating DKD phenotypes (i.e., DKD group, DKD5 group, and DKD mice, **Table 3**). Among them, *Muribaculaceae* does not even exist in the subjects with more relieved DKD phenotypes (i.e., DKD 1&2 and 3 in patients, healthy control, and FMT mice), but was accumulated in the subjects with more serious DKD phenotypes (DKD5), even though there is no statistical significance when compared with DKD5 and other DKD stages.

**Table 3:** The potentially harmful significantly accumulated OTUs shared by DKD when compared with DMHC, DKD5 when compared with other DKD stages, and DKD mice when compared with Con and FMT mice.

OTUs	DKD V.S. DMHC				DKD5 V.S. Other stages							DKD rats V.S. Con & FMT rats			
	DKD mean abundance	DMHC mean abundance	P-Value	Significance mark	DKD1&2 mean abundance	DKD3 mean abundance	DKD4 mean abundance	DKD5 mean abundance	P-value	Significance mark	Con mean abundance	DKD mean abundance	FMT mean abundance	P-value	
OTU83 (Muribaculaceae)	0.00190495	9.98E-04	0.00211266	**	0	0	8.95E-07	2.10E-04	0.125067126		0	0.011061875	3.57E-06	<0.001	***
OTU303 (Ruminococcaceae_uncultured)	1.93E-04	2.97E-05	<0.001	***	1.72E-04	5.06E-06	1.84E-04	3.88E-04	0.008703768	**	1.16E-05	7.23E-04	1.97E-04	<0.001	***
OTU105 (Flavobacteriaceae_uncultured)	1.23E-05	2.45E-06	0.00121705	**	0	7.44E-06	4.70E-06	4.99E-05	0.008009472	**	2.74E-04	0.0021485	0.001269857	0.00226052	**
OTU154 (Subdoligranulum)	1.19E-04	2.09E-06	<0.001	***	1.75E-05	0	3.44E-05	5.77E-04	0.010933308	*	0	0.002816375	0	0.00126895	**
OTU251 (Oscillospiraceae_UCG-002)	0.015745408	0.006829418	0.00387515	**	0.007366875	0.004258313	0.012553237	0.04041675	0.009132116	**	0	0.002146375	0	0.00503556	**
OTU241 (Lachnospiraceae_unclassified)	3.91E-05	5.72E-06	<0.001	***	0	1.20E-05	4.39E-05	5.83E-05	<0.001	***	0	0.001966875	0	0.00503556	**
OTU246 (Oscillospirales_UCG-010)	6.36E-05	2.66E-05	0.01900196	*	0	1.31E-06	3.72E-05	2.39E-04	0.003385615	**	5.43E-06	7.25E-04	2.64E-04	0.00275607	**
OTU176 (Christensenellaceae_R-7_group)	0.001168783	1.28E-04	<0.001	***	1.30E-04	0	0.001441171	0.0014841	0.001131849	**	0	5.87E-04	4.63E-05	0.00907164	**
OTU483 (Lachnospiraceae_unclassified)	2.66E-04	1.93E-05	<0.001	***	8.08E-05	6.73E-05	1.23E-04	0.0010449	<0.001	***	0	1.37E-04	0	0.00126895	**
OTU529 (Christensenellaceae_R-7_group)	5.88E-05	2.04E-05	<0.001	***	1.01E-05	2.18E-05	4.32E-05	1.67E-04	0.016998359	*	0	4.00E-05	0	0.00503556	**
OTU233 (Ruminococcaceae_unclassified)	1.31E-05	3.02E-07	<0.001	***	0	0	6.92E-06	5.20E-05	0.006485023	**	0	0.001572	1.67E-05	0.01628199	*
OTU202 (Ruminococcaceae_unclassified)	2.07E-05	9.35E-06	0.00189313	**	0	0	2.04E-05	4.65E-05	0.005952553	**	1.30E-05	3.74E-04	2.42E-04	0.02650222	*
OTU140 ([Eubacterium]_siraeum_group)	0.003044842	2.78E-04	<0.001	***	2.41E-05	9.68E-04	0.003240105	0.005173	<0.001	***	1.77E-04	2.18E-04	0	0.04239545	*
OTU313 (Butyricicoccaceae_UCG-009)	1.28E-05	1.29E-07	<0.001	***	1.78E-05	1.16E-05	3.83E-06	4.60E-05	0.040648438	*	4.43E-06	2.70E-04	7.00E-05	0.03125367	*

OTU, Operational Taxonomy Units; DKD, Diabetic Kidney Disease; DMHC, Diabetes Mellitus and Healthy control; Con, healthy control; FMT, Fecal Microbiota Transplantation; *P<0.05, **P<0.01, ***P<0.001.

To reveal the potential DKD-exacerbating effects, the OTUs above underwent Spearman correlation analysis with clinical indices of the patient with different DKD stages. *[Eubacterium]_siraeum_group* and *Christensenellaceae_R-7_group* were both negatively correlated with eGFR and positively correlated with 24-h urine protein, serum creatine, and urea (**Figure 5I**, **Table S25**). *Ruminococcaceae_uncultured* was negatively correlated with eGFR and positively correlated with serum creatinine and urea. *Oscillospiraceae_UCG-002* was positively correlated with 24-h urine protein and serum urea. *Lachnospiraceae_unclassified* was positively correlated with serum urea and creatinine and negatively correlated with eGFR. All of the OTUs mentioned above showed the obvious DKD phenotype-exacerbating effects.

### FMT alters metabolomic features of DKD state *via* influencing gut microbiota

**Figure 8:**
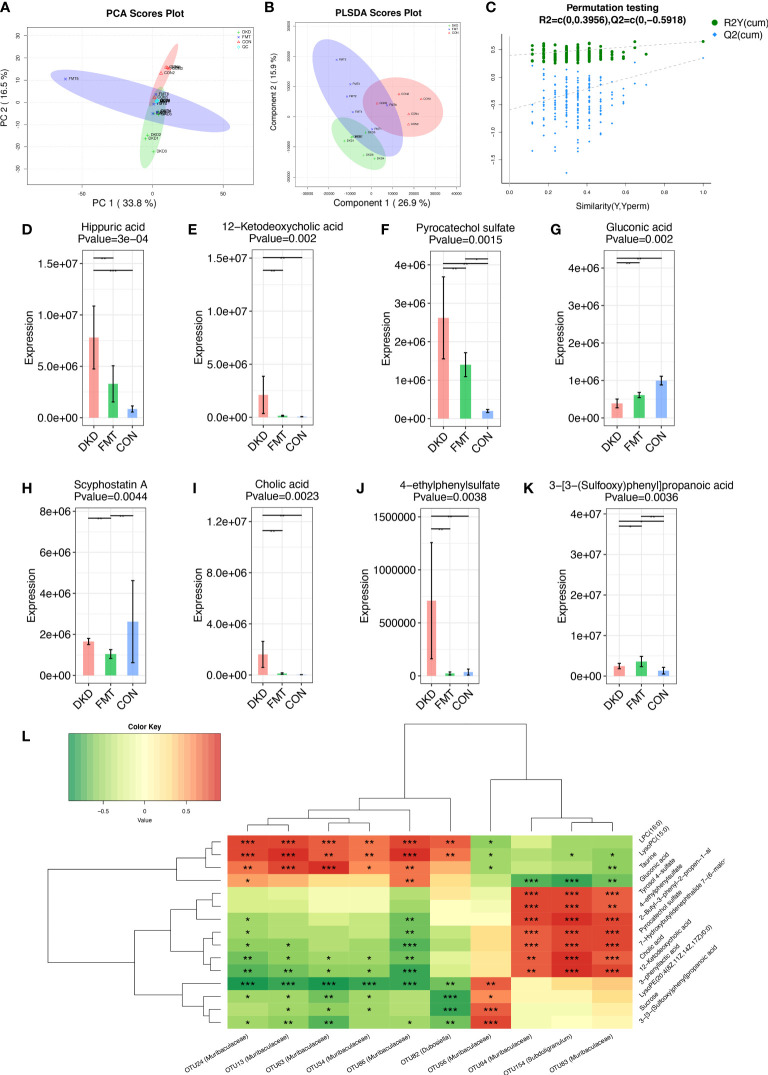
Metabolic alterations of mouse models based on untargeted metabolomic detection and Spearman’s correlation analysis between important differential metabolites and key OTUs. PCA scores plots **(A)** and PLS-DA scores plots **(B)** validated serum metabolites disparity between three groups. Scatter plots of the statistical validations obtained by 200’s permutation tests **(C)**. The boxplots showing the comparison of the relative expression level of 8 important differential metabolites among three groups **(D–K)**. The Spearman’s correlation relationship between important differential metabolites and key OTUs were presented as heatmap **(L)**. PCA, principle component analysis; PLSDA, Partial Least-Squares Discrimination Analysis. *p<0.05; **p<0.01; ***p<0.001.

Considering we uncovered that decreasing the potentially harmful microbial community by FMT can efficiently alleviate DKD phenotypes, we further employed untargeted serum metabolomic analysis to globally describe the metabolic features of DKD and FMT mice, aiming to discover the underlying effects of microbiome on metabolism. We identified a total of 276 and 372 metabolites in positive and negative ion mode, respectively (**Table S26**, **S27**). PCA and PLS-DA analysis revealed significant disparities between the FMT and DKD group (accumulative *R* ^2^ *X* = 0.503, **Figure 8A**; *R* ^2^ *Y* = 0.699, Q2 = 0.291, **Figure 8B**, respectively). In 200’s permutation test, all *R* ^2^ and Q2 values of permutated models were worse than the original model, indicating a better prediction ability and reliability of this model (**Figure 8C**). Therefore, we revealed that the serum metabolite profiles of the FMT group has dramatically altered compared with the DKD group. Then, we selected the metabolites that were significantly different (*p*-value < 0.05) between DKD and FMT mice group and defined those metabolites whose VIP value > 1.0 as important differential metabolites. After healthy FMT, the serum level hippuric acid, 12-hetodeoxycholic acid, pyrocatechol sulfate, scyphostatin A, cholic acid, and 4-ethylphenylsulfate were significantly decreased when compared with the DKD group (**Figures 8D–K**). As several important differential metabolites (e.g., hippuric acid and cholic acid) are considered as microbiota-derived uremic solutes, we then implemented the microbiota–metabolome combining Spearman correlation analysis to preliminary construct the potential relationship between selected potentially harmful microbes and metabolites. As shown by **Figure 8L**, serum levels of cholic acid, pyrocatechol sulfate, and 4-ethylphenylsulfate acid were positively correlated with the abundance of *subdoligranulum* and *muribaculaceae*, which are both accumulated in the subjects with more exacerbating DKD phenotypes.

## Discussion

In our study, we demonstrated marked dysbiosis and decreased diversity in gut microbial profile of DKD compared with non-DKD population (including healthy individuals and DM patients) using 16S rRNA sequencing. Meanwhile, we constructed the clinical classifier that can relatively diagnose DKD from general populations efficiently, hopefully providing the more convenient and noninvasive method in real clinical practice. Furthermore, the key OTUs that we selected to construct the DKD clinical classifier were all closely correlated with DKD-related clinical indices as results showed, also providing firm theoretic underpinnings for the clinical application. Through animal experiments, we discovered the potentially harmful microbes accumulated in DKD mice that were also accumulated in DKD patients. Additionally, we also constructed the underlying connection between gut microbiota and serum metabolome in mice, providing the reference for further research about DKD pathogenesis from a metabolic perspective.

Emerging lines of evidence have demonstrated that the development of many chronic diseases is associated with abnormal gut microbiota (26–28). Gut microbial composition is a susceptibility factor for individuals with a predisposition to develop nephropathy, such as patients with DM (29). In patients with chronic kidney disease, gut microbial composition was also found to be significantly altered (30, 31). Our previous study reported that gut microbial profile was unique in DKD patients and quite different from that in membranous nephropathy patients (32). Moreover, we also found significant gut dysbiosis in DKD patients when compared with the non-DKD population, which was consistent with a recent research (33).

A typical altered gut microbiome could be a non-invasive biomarker for disease diagnosis (34, 35). In our study, 10 obtained OTUs as biomarkers achieved good diagnostic capability for DKD. Most species of *Lactobacillus* were recognized as beneficial bacteria and treatment target in DM (36, 37). *Lachnospiraceae UCG-004* was found to be the potential dominant bacteria and biomarker in reducing the blood glucose and insulin resistance of DM mice (38). Many species in *Faecalibacterium* such as *Faecalibacterium prausnitzii* was discovered to be related to gut barrier integrity and inflammation in DM (39).

We attempted to further clarify the potential role of microbiota on DKD exacerbation. Based on the clinical diagnostic criteria of DKD stages, the DKD patients were stratified into four groups (DKD stage 1&2, stage 3, stage 4, and stage 5). Their 16S rRNA sequencing data of gut microbiota were analyzed. Significant disparity of gut microbiota was also observed among four groups. However, the diversity of gut microbiota was significantly increased during DKD exacerbation, while the microbial diversity was significantly decreased in DKD patients compared with non-DKD populations. We hypothesized that abnormal increased diversity may be due to the types and richness of some potential pathogens that may gradually expand and dominate the community during DKD deterioration. In Tao’s study, increased diversity of the gut microbiome was also observed in 14 DNs compared with 14 DMs, which was primarily due to the richness of harmful bacteria especially *Escherichia-Shigella* (16). A recent study divided DKD patients into two groups according to their serum creatine and identified 11 different intestinal floras between two groups (40). In our research, we revealed that 16 genera bacteria were significantly enriched in DKD patients and accumulate with the DKD progression, implying the dynamic changes in gut microbiota from the onset to the progression of DKD, and the accumulation of these 16 genera may be involved in the DKD pathogenesis and exacerbation. Among them, *[Eubacterium]_siraeum_group, Ruminococcaceae_incertae_sedis*, and *Acetanaerobacterium* were the three most significantly accumulated genera in DKD5. According to the previous reports, *[Eubacterium]_siraeum_group* was positively associated with increased systolic and diastolic blood pressure and HDL level (17, 41). *Moryella* was also reported to be accumulated in obese mice when compared with obese mice receiving resveratrol (21).

We then conducted the animal experiment to repetitively confirm the findings that we just uncovered during our gut microbiota analysis of the large DKD cohort and find the potential targeted harmful bacteria. We successfully developed the DKD mouse models and analyzed the intestinal microbial profiles. The gut microbiota of DKD mice were significantly dysbiotic when compared with the control mice and the diversity was dramatically decreased, which is consistent with our human study. We also constructed the DKD mice that were transplanted with the feces from control mice, trying to reverse the normal state of gut microbiota. The DKD phenotypes were generally relieved after 14 days of fecal microbiota transplantation, suggesting that the dysbiotic community does play a role in DKD pathogenesis and exacerbation. Then, we collectively selected the key OTUs that are dramatically accumulated in both human and mice with more exacerbating DKD phenotypes (i.e., the DKD and DKD5 patients and DKD mice). We successfully selected the common shared 14 OTUs that were closely correlated with clinical renal indices. Furthermore, after fecal microbiota transplantation, the abundance of all 19 OTUs was significantly depressed and the phenotypes are relieved.

Increasing lines of evidence have demonstrated the association of gut microbiota with many chronic metabolic diseases including type 2 DM, nonalcoholic fatty liver disease, and obesity. Alteration in the gut microbiome structure has been detected in CKD patients, following the expansion of pathogenic microbes, thereby increasing the synthesis of uremic toxins (22, 42). The intestinal flora produced amines, indoles, and phenols by fermenting undigested proteins and peptides reached in the colon. Then, the microbiota-produced intermediates of uremic toxins will be absorbed and accumulated in the serum of CKD patients, potentially aggravating the disease. The uremic toxins were cytotoxic, affected biological functions, and exerted pathological impact on kidney, blood vessels, and the immune system (43). Therefore, we further clarify the underlying connection between gut microbiota and serum metabolites. Combining with the untargeted metabolomic detection technology of mice serum samples, we globally delineated the metabolic profiles of DKD and healthy fecal microbiota transplanted mice. We revealed that metabolic disturbance existing in DKD and the FMT method can significantly alter the metabolic features of DKD. Through the combined correlation analysis, we also found the potential connection between the gut microbiota and metabolome. Considering we uncovered the fact that FMT can alleviate the clinical phenotypes of DKD, we specifically revealed several dramatically changed microbiota metabolites that are correlated with the significantly changed microbes after FMT, hoping to construct the relationship of gut microbiota–metabolome–DKD alleviation and inspire further research.

Compared with healthy mice, hippuric acid is significantly accumulated in DKD mice, which is consistent with a previous study (44). Hippuric acid is involved in phenylalanine metabolism and considered as a microbiota-derived uremic solute and related to chronic kidney disease (45). The clinical study has demonstrated that the plasma level of hippuric acid is shown to be elevated in hemodialysis patients with chronic renal failure compared with healthy controls and hospital patients without kidney disease (46). Surprisingly, healthy microbiota transplantation can significantly decrease the serum hippuric acid level, which is comparable with the healthy control mice, suggesting that remodeling healthy intestinal microbial community may decrease some uremic toxins specifically and improve DKD phenotypes. Meanwhile, cholic acid was also acknowledged as uremic toxins and has been confirmed to be elevated in the serum of CKD mice (47). Our study revealed that the serum level of cholic acid was increased in DKD mice and was positively correlated with the abundance of *muribaculaceae* and *subdoligranulum*. *Subdoligranulum* was also reported to be positively correlated with 24-h urine protein and might be a detrimental factor in DN (48). Furthermore, all OTUs mentioned above were significantly accumulated in DKD mice when compared with healthy mice. However, healthy FMT can recover the abundance of all OTUs mentioned above, which were comparable to those of healthy controls. We speculated reasonably that healthy FMT can decrease the accumulated potentially cholic acid-producing harmful bacteria in DKD mice, decrease the production of jeopardizing cholic acid, and further improve the DKD phenotypes. Nowadays, the underlying connection between intestinal microbe and metabolites has become a hot issue. *Prevotellaceae_NK3B31_group* was a probiotic with a significant decrease in CKD patients. Studies have shown that *Prevotellaceae_NK3B31_group* was correlated with anti-inflammation and the level of trimethylamine-N-oxide (TMAO) (49, 50). Subsequent studies, such as single microbe transplantation, are needed to discover the potential metabolic processes between the targeted microbes and uremic toxins.

## Conclusion

**Figure 9:**
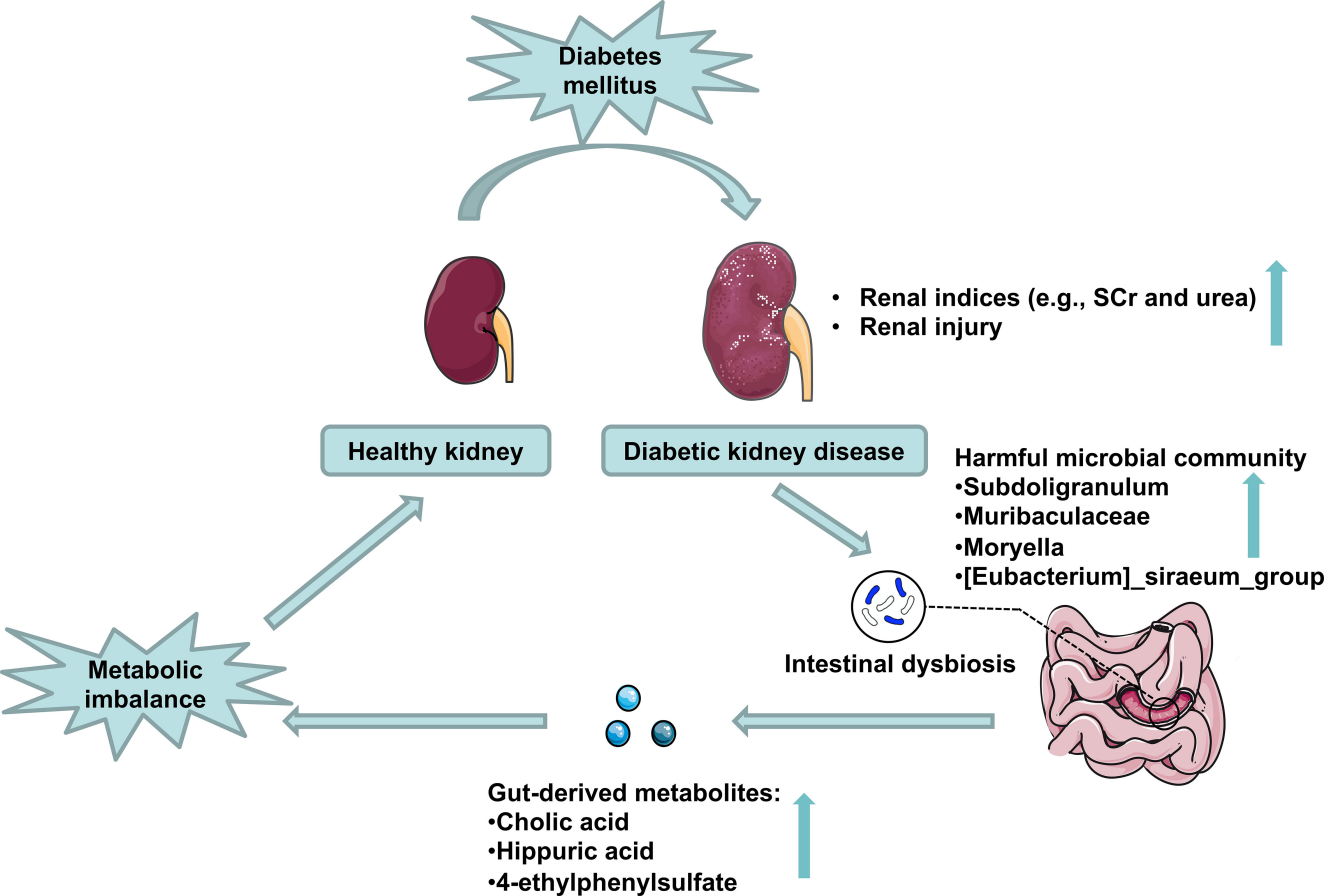
Schematic representation of the potential mechanisms of gut microbiota on DKD exacerbation. DKD induces intestinal dysbiosis, which presents as harmful microbial community accumulation, further increasing gut-derived injurious metabolites production, which disturbs host serum metabolomics and exacerbates DKD phenotypes.

The fecal microbial community was altered markedly in DKD. The gut microbiome could be used as a biomarker in the diagnosis of DKD. Combining the fecal analysis of both human and animal models validated and selected the targeted accumulated harmful pathogens. Healthy FMT can partially recover the microbial community and relieve DKD phenotypes *via* influencing pathogens’ effects on DKD mice’s metabolism (**Figure 9**).

## Data availability statement

The data presented in the study are deposited in the NCBI (SRA number: PRJNA881044, PRJNA756402, PRJNA660302 and PRJNA876080)) and Metabolights repository (https://www.ebi.ac.uk/metabolights/), accession number MTBLS6040 (www.ebi.ac.uk/metabolights/MTBLS6040).

## Ethics statement

This study was reviewed and approved by First Affiliated Hospital, School of Medicine, Zhengzhou University (2019-KY-361 and 2021-KY-0162). The patients/participants provided their written informed consent to participate in this study. The animal study was reviewed and approved by Ethical Committee of Experimental Animal Care of First Affiliated Hospital of Zhengzhou University (2021-KY-0162).

## Author contributions

ZZ and JS provided financial support. JS and ZR help to design this study. RG, WC, YZ, PW, WY, XZ and TW contribute to rat experiment. YD, JZ, SD collected samples and clinical data for this project. YZ and JX supervised clinical and experimental data. JS, RG and WC completed the draft of this manuscript. JS and ZR supervised the study and revised manuscript. All authors contributed to the article and approved the submitted version.

## Funding

We thank all volunteers for providing samples for our study. This work was supported by the National Natural Science Foundation of China (Grant Nos. 81873611, 8217033050, and U2004121), the 2020 Key Project of Medical Science and Technology to Shang Jin, and the National Key Research and Development Program of China (2018YFC2000501). All of the human samples were obtained from the Biobank of The First Affiliated Hospital of Zhengzhou University and National Human Genetic Resources Sharing Service Platform (Grant No. 2005DKA21300).

## Conflict of interest

The authors declare that the research was conducted in the absence of any commercial or financial relationships that could be construed as a potential conflict of interest.

## Publisher’s note

All claims expressed in this article are solely those of the authors and do not necessarily represent those of their affiliated organizations, or those of the publisher, the editors and the reviewers. Any product that may be evaluated in this article, or claim that may be made by its manufacturer, is not guaranteed or endorsed by the publisher.

## Supplementary material

Supplementary Figure 1 Quality control of 16S rRNA sequencing data. **(A)** species accumulation curve indicated that number of samples had approached saturation in DKD (n=120), DM (n=92) and Con group (n=140). **(B)** Rarefaction analysis showed that enough depth of 16S rRNA sequencing data had been achieved. **(C)** Rank-Abundance curve mainly explained microbial OTU-based evenness and richness in discovery group. **(D)** Shannon-Wiener curve estimated microbial OTU-based diversity in discovery group. OTU, operational taxonomy units; DKD, diabetic kidney disease; DM, diabetes mellitus; Con, healthy controls.

Supplementary Figure 2 Alpha and Beta diversity between DKD and non-DKD group. Chao1/Shannon indices **(A, B)** were used to assess alpha diversity. NMDS Calculated by Bray–Curtis technique **(C)** and unweighted UniFrac distances **(E).** Unweighted UniFrac distances were used to assess significance by PCoA analysis **(E, F)**. PCoA, principal coordinate analysis; PC, principal component, PC1 and PC3. NMDS, non-metric multidimensional scaling analysis; Adonis, permutational/nonparametric multivariate analysis of variance.

Supplementary Figure 3 Average microbial composition between DKD and non-DKD group were shown at class **(A)**, family **(B)** or order **(C)** levels. Microbial comparison among four groups at the class **(D)**, family **(E)** or order **(F)** level through Kruskal-Wallis test.

Supplementary Figure 4 Phylogenetic composition and comparison from phylum to genus level were shown in pattern of cladogram **(A)**. LEfSe comparison integrated with bacteria on genus level between DN and MN **(B)**. The length of histogram represented variable importance. LDA score > 2.0, P < 0.05. Spearman’s correlation analysis between crucial clinical parameter and key OTUs when compared DKD patients with non-DKD populations **(C)**. LEfSe, linear discriminate analysis and effect size.

Supplementary Figure 5 Average microbial composition among the DKD1&2, DKD3, DKD4 and DKD5 group were shown class **(A)**, order **(B)** or family **(C)** level. Microbial comparison among four groups at the class **(D)**, order **(E)** or family **(F)** level through Kruskal-Wallis test.

Supplementary Table 1 Comparison of α-diversity in discovery cohort (DKD=120 and DMHC=232)

Supplementary Table 2 Separating distribution of community between DKD and DMHC groups in unweighted UniFrac distance.

Supplementary Table 3 Distribution of key OTUs (n=35) with relative abundance>0.003% in all samples

Supplementary Table 4 Microbial composition and comparison between DKD and DMHC groups at the phylum level

Supplementary Table 5 Microbial composition and comparison between DKD and DMHC groups at the class level

Supplementary Table 6 Microbial composition and comparison between DKD and DMHC groups at the order level

Supplementary Table 7 Microbial composition and comparison between DKD and DMHC groups at the family level

Supplementary Table 8 Microbial composition and comparison among three groups at the genus level

Supplementary Table 9 LDA integrated LEfSe analysis showed clear distinction of gut microbiome between DKD and DMHC groups.

Supplementary Table 10 Genus-level annotation of identification markers for classification of DKDs and non-DKDs.

Supplementary Table 11 Spearman correlation analysis of optimal OTUs and clinical variables

Supplementary Table 12 POD index of each sample in discovery phase

Supplementary Table 13 POD index of each sample in validation phase

Supplementary Table 14 Comparison of α-diversity among various DKD stages (DKD1&2 = 12, DKD3 = 26, DKD4 = 112 and DKD5 = 30).

Supplementary Table 15 Operational taxonomic unit (OTU) profiles among all samples in various DKD groups.

Supplementary Table 16 Separating distribution of community among various DKD stages in unweighted UniFrac distance.

Supplementary Table 17 Microbial composition and comparison among different DKD stages at the phylum level

Supplementary Table 18 Microbial composition and comparison among different DKD stages at the class level

Supplementary Table 19 Microbial composition and comparison among different DKD stages at the order level

Supplementary Table 20 Microbial composition and comparison among different DKD stages at the family level

Supplementary Table 21 Microbial composition and comparison among different DKD stages at the genus level

Supplementary Table 22 Separating distribution of community among control, DKD and FMT mice in unweighted UniFrac distance.

Supplementary Table 23 Microbial composition and comparison among control, DKD and FMT mice at the genus level

Supplementary Table 24 Spearman correlation analysis of optimal OTUs and clinical variables

Supplementary Table 25 Positive metabolites detected in serum samples from control, DKD and FMT mice.

Supplementary Table 26 Negative metabolites detected in serum samples from control, DKD and FMT mice.
