# Supplementary material for: The harmful intestinal microbial community accumulates during DKD exacerbation and microbiome–metabolome combined validation in a mouse model
Source: Front Endocrinol (Lausanne). 2022 Dec 19;13:964389. doi: 10.3389/fendo.2022.964389 (PMC9806430; doi:10.3389/fendo.2022.964389)
Supplement: Supplementary file 1 [file DataSheet_1.docx]

**Supplementary file 1**

Beads-beating method was used to lyse bacterial cell membrane. We implemented PCR to amplify the 16s rRNA, the highly conservative V3-V4 region, so that we got puriﬁed amplicons for Miseq sequencing. PCR was conducted using the following program: 3 min of denaturation at 95℃, 21 cycles of 0.5 min at 94℃ (denaturation), 0.5 min for annealing at 58℃, 0.5 min at 72℃, and 5 min at 72℃ for a final extension. PCR was performed in a 20µl reaction system containing 4µl 5×Fastpfu buffffer, 2µl 2.5mM deoxynucleotide triphosphates (dNTPs), 0.8µl of each primer (5µM), 0.4µl TransStart Fastpfu DNA polymerase (TransGen Biotech, Beijing, China), and 10ng template DNA. The PCR products were detected on a 2% agarose gel, and the band was extracted and purified using the AxyPrepDNA gel (Axygen, CA, USA) and PCR clean-up system. The purified PCR products were mixed. Sequencing was performed on an Illumina MiSeq platform according to the standard protocols of the Shanghai Mobio Biomedical Technology Co. Ltd., China.

**Supplementary file 2**

Bacterial richness and diversity were estimated separately by Chao/Observed OTU and Shannon/Simpson indices in our study. Mann-Whitney U test was used to compare OTU differences of two groups. Differences of three groups were compared using Kruskal-Wallis test. Bray–Curtis dissimilarity and (un)weighted UniFrac distances were calculated in QIIME to assess beta diversity. In addition, principal coordinate analysis (PCoA) and non-metric multidimensional scaling analysis (NMDS) were performed to aid interpretation of bacterial distribution among three groups. Correspondingly, we used permutational multivariate analysis of variance (PERMANOVA) and analysis of similarities (ANOSIM) to evaluate the statistical significance of differences among three groups. Significantly different genera in discovery cohort were determined using the linear discriminant analysis (LDA) effect size (LEfSe). Significant taxonomic distribution between DN and MN was shown with LDA values>2.0 and p-value<0.05. Correlation between fecal microbiota and clinical variables was calculated by Spearman correlation analysis and showed in the pattern of heatmap. Phylogenetic Investigation of Communities by Reconstruction of Unobserved states (PICRUSt) was used to predict markedly enriched KEGG metabolic pathways in different disease status (LDA scores>2.0 and p-value<0.05).

By five repeats of five-fold cross-validation, we got the minimum cross-validation error rate (CV error rate) and defined this value as the cutoff. OTU set with a CV error rate less than cutoff and the minimum number was considered as the optimal set. Based on the optimal set, we performed receiver operating characteristic (ROC) curve construction for discovery cohort and validation cohort.

**Supplementary file 3**

The fresh feces of healthy C57/BL6 mice were collected and weighed, then diluted with normal saline whose volume was adjusted so that the concentration of fecal suspension was approximately 120 mg/ml. Then the fecal suspension was filtered with a strainer to remove large particles. The filtrate was collected and mixed with glycerol to adjust the concentration of glycerol to 20% and placed at - 80 ℃ for storage.

All mice were housed under standard specific pathogen-free (SPF) conditions with a 12 h light/dark cycle at 22~24° C and allowed to eat a standard diet and drink randomly.

Intraperitoneally injected streptozotocin (STZ) together with high fat diet (HFD) feeding was used to induce DKD. We verified DKD model construction based on the blood glucose, urine protein and renal histopathological characteristics.

All mice were weighed once a week. The blood glucose level of caudal vein was measured once a week using electronic glucose meter. The feces, urine and blood samples of each mice were collected once a week from obtained. All the urine and feces were collected freshly and immediately frozen at −4℃. We collected the plasma after a centrifugation at 4℃, 4000 rpm for 10 min. All of samples above were transferred to a -80℃ environment within 2 hours for further analysis. The feces were subjected to 16S rRNA sequencing to describe the gut microbiota profiles of 4 groups. The plasma were subjected to untargeted metabolomic analysis using UPLC-MS to delineate the serum metabolic profiles of 4 groups. The urine was collected from all 4 groups for total urinary protein/ urinary creatinine (T/Cr) test to evaluate the severity of DKD.

After DKD induced by 3-month duration of intraperitoneal STZ injection together with HFD, the broad-spectrum antibiotic cocktail was implemented to FMT group for another 14-day course. The antimicrobial solution was made up of Ampicillin 0.1g/L, Vancomycin 0.5g/L, Neomycin 1 g/L, Metronidazole 1g/L, replacing purified water for daily water intake.

After clearance of gut microbiota, the fecal microbial solution produced from healthy mice’s feces were transplanted to FMT group by gavage for another 14 days to reconstruct the gut microbiota of healthy donors. One week after the end of gavage, all the mice were sacrificed. All kidneys were collected for further histopathological evaluation.

**Supplementary file 4**

Step of HE staining: (1) Paraffin sections conventional dewaxing to water. (2) Hematoxylin staining for 3 min. (3) Wash with water and separate with 1% hydrochloric acid alcohol for 10s. (4) Wash with water and stain with 1% eosin for 10 min. (5) Wash with water and dehydrate with gradient alcohol. (6) Transparency using xylene and seal with neutral gum. (7) Microscope observation and photography.

Step of PAS staining: (1) Conventional dehydration of 10% formalin. (2) Paraffin sections conventional dewaxing to water. (3) Rinse with tap water for 2-3min, and then soak twice with distilled water. (4) Place into periodic acid solution for 8 min at room temperature. (5) Rinse once with tap water and soak twice with distilled water. (6) Place into Schiff region in the shade at room temperature, and dip it for 11 min. (7) Rinse with tap water for 10 min. (8) Place into hematoxylin staining solution for 1-2min. (9) Place into acidic ethanol solution for 2-5 s. (10) After washing with tap water for 10-15min, change the double distilled water to make it turn blue. (11) Dehydrate with gradient alcohol. Transparency using xylene and seal with neutral gum. (12) Microscopic observation and photography.

Step of Masson staining: (1) Paraffin section dewaxing to water. (2) Wash with tap water and distilled water in turn. (3) Stain with Regaud hematoxylin for 5-10 minutes. (4) Wash thoroughly. If it is over stained, differentiate into hydrochloric acid and alcohol. (5) Wash with distilled water. (6) Stain with Masson fuchsin acid fuchsin solution for 5-10 minutes. (7) Soak in 2% glacial acetic acid solution for a while. (8) Differentiate with 1% molybdophosphoric acid solution for 3-5 minutes. (9) Stain with aniline blue for 5 minutes without washing. (10) Soak in 0.2% glacial acetic acid solution for a while. (11) Wash in 95% alcohol and anhydrous alcohol. Transparency using xylene and seal with neutral gum. (12) Microscope observation and photography.

The ultrastructure of human renal proximal tubule cells was observed with TEM to assess autophagy. After treat- ment, the cells were harvested and fixed in 0.1 M cacodylate buffer containing 2.5% glutaraldehyde (Sigma) and post- fixed in 1% osmium tetroxide. After treatment with 0.5% tannic acid and 1% sodium sulfate, the cells were cleared in 2-hydroxypropyl methacrylate, embedded in Ultracut (Leica, Wetzlar, Germany), sliced into sections with a thickness of 60 nm and stained with uranyl acetate and lead citrate. The ultrathin sections were then examined using a JEM-1230 TEM (JEOL, Tokyo, Japan

**Supplementary file 5**

The serum samples of DKD, FMT and healthy control mice were collected, temporarily conserved in ice packs and immediately sent to the Biobank of The First Affiliated Hospital of Zhengzhou University for cold storage at -80 ˚C environment within 2 hours for further untargeted metabolomic analysis. The methanol, acetonitrile, and water were all obtained from Fisher Chemical (Shanghai, China). Formic acid was purchased from CNW. 2-Propanol was obtained from Merck. 2-Chloro-L-Pheylalanine was from Adamas-beta. The centrifuge 5424R and 5430R were both purchased from Eppendorf (Shanghai, China). UHPLC liquid chromatography system (Vanquish Horizon System) and mass spectrometer (Q-Exactive HF-X) were both purchased from Thermo Scientific (Shanghai, China). The samples were pre-processed to remove proteins and impurities. We transferred 100 µL sample into a 1.5 mL centrifuge tube, added 400 µL methanol containing 0.02 mg/mL internal standard (L-2-chlorophenylalanine). After vortexing and mixing for 30s, the samples underwent low-temperature ultrasonic extraction (5℃, 40KHz) and were frozen at -20 ˚C for half an hour, respectively. Then centrifuged samples for 15 min (13,000 g, 4 ˚C), supernatants were collected and transferred into injection sample vials for further computer analysis. During the test, the QC samples were evenly and randomly distributed in the injection process.

A Vanquish Horizon ultra-high performance liquid chromatography system (Thermo Scientific) was equipped with ACQUITY UPLC HSS T3 column (100 mm × 2.1 mm i.d., 1.8 µm; Waters, Milford, USA). The binary gradient elation system consisted of mobile phase (A), which is composed of 95% water and 5% acetonitrile (containing 0.1% Formic acid), and (B), which is composed of 47.5% acetonitrile, 47.5% isopropanol and 5% water (containing 0.1% Formic acid). The separation was achieved using following gradient: 0-100% B over 0-5.5 min, the composition was held at 100% B at 5.5-7.4 min, then 7.4-7.8 min, 100% to 0 B, and 7.8-10 min holding at 0 B. The flow rate was 0.4 mL/min, and the column temperature was 40 ˚C, the injection volume was 2 µL. Mass spectrometry was performed on a Q-Exactive HF-X system (Thermo Scientific). The mass range was from m/z 70-1050. The resolution was set at 60 000 for the full MS scans and 7500 for MS2 scans. The samples were ionized by electrospray and the mass spectrometry operated as follows: spray voltage, 3500 V (positive) and 3500 V (negative); sheath gas flow rate, 50 arbitrary units; auxiliary gas flow rate, 13 arbitrary units; capillary temperature, 325 ˚C.

The original metabolomic data was processed using the Progenesis QI (WaterCorporation, Milford, USA), which produced a matrix features with retention time, peak area, mass-to-charge ratio and identification information. All variables were normalized to the total peak area of each sample. All metabolites were identified by MS and MS/MS fragment through Progenesis QI (WaterCorporation, Milford, USA) with several mainstream public databases (http://www.hmdb.ca/, https://metlin.scripps.edu/). Afterwards, the ProgenesisQI (WaterCorporation, Milford, USA) was used to search and identify the characteristic peaks. We set MS mass error as less than 10 ppm, matched the MS and MS/MS mass spectrum information with the metabolic database, then the metabolites were identified based on the secondary mass spectrometry matching score. The main databases used for metabolites identification are several mainstream public databases (http://www.hmdb.ca/, https://metlin.scripps.edu/). Principle component analysis (PCA) and Orthogonal Partial Least-Squares Discrimination Analysis (OPLS-DA) were performed to identify the discrimination of variables. Permutation testing was used to evaluate the accuracy of PLS/OPLS-DA. Based on OPLS-DA analysis, the metabolites with variable importance in projection (VIP) > 1 are recognized as important variables. VIP represents the ability to extract variables of differentiation among groups. Important differential metabolites were defined as those with VIP > 1.0 obtained from OPLS-DA and adjusted P values <0.05. Based on HMDB, KEGG and LIPID MAPS databases, all important differential metabolites were annotated to specific pathways and classified based on pathways’ function. KEGG pathway topology was applied to evaluate the extent of important differential metabolites’ influence on their functional pathways.

Data collected from biochemical assay were expressed as mean ± SEM. Statistical analyses were performed using SPSS 23.0 software. Comparisons between groups were measured by Student’s T test. P values < 0.05 were considered statistically signiﬁcant.
