# Supplementary figures and images for: The harmful intestinal microbial community accumulates during DKD exacerbation and microbiome–metabolome combined validation in a mouse model
Source: Front Endocrinol (Lausanne). 2022 Dec 19;13:964389. doi: 10.3389/fendo.2022.964389 (PMC9806430; doi:10.3389/fendo.2022.964389)

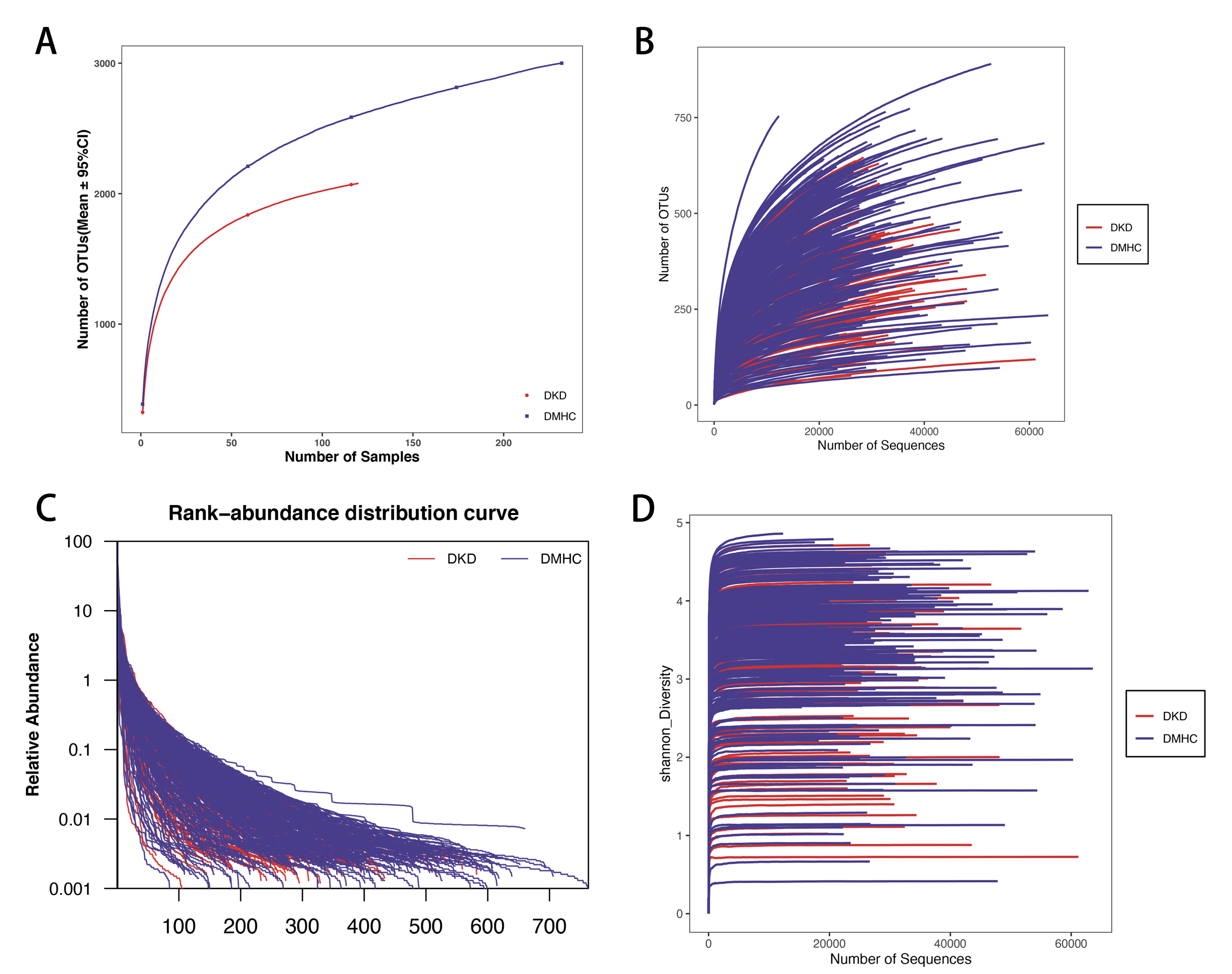

Supplement: Supplementary Figure 1 — Quality control of 16S rRNA sequencing data. (A) species accumulation curve indicated that number of samples had approached saturation in DKD (n=120), DM (n=92) and Con group (n=140). (B) Rarefaction analysis showed that enough depth of 16S rRNA sequencing data had been achieved. (C) Rank-Abundance curve mainly explained microbial OTU-based evenness and richness in discovery group. (D) Shannon-Wiener curve estimated microbial OTU-based diversity in discovery group. OTU, operational taxonomy units; DKD, diabetic kidney disease; DM, diabetes mellitus; Con, healthy controls. [file Image_1.tif]

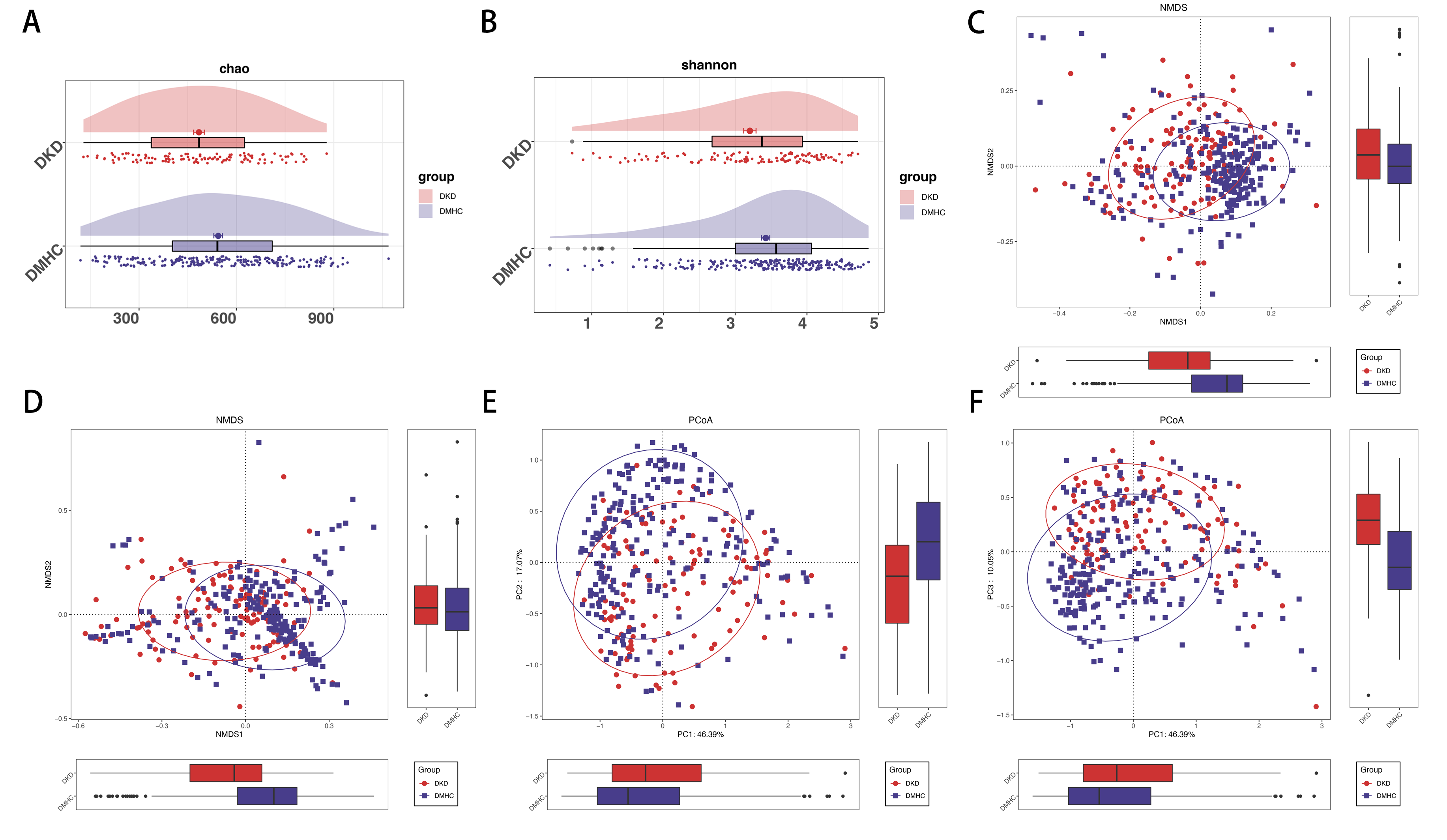

Supplement: Supplementary Figure 2 — Alpha and Beta diversity between DKD and non-DKD group. Chao1/Shannon indices (A, B) were used to assess alpha diversity. NMDS Calculated by Bray–Curtis technique (C) and unweighted UniFrac distances (E). Unweighted UniFrac distances were used to assess significance by PCoA analysis (E, F). PCoA, principal coordinate analysis; PC, principal component, PC1 and PC3. NMDS, non-metric multidimensional scaling analysis; Adonis, permutational/nonparametric multivariate analysis of variance. [file Image_2.tif]

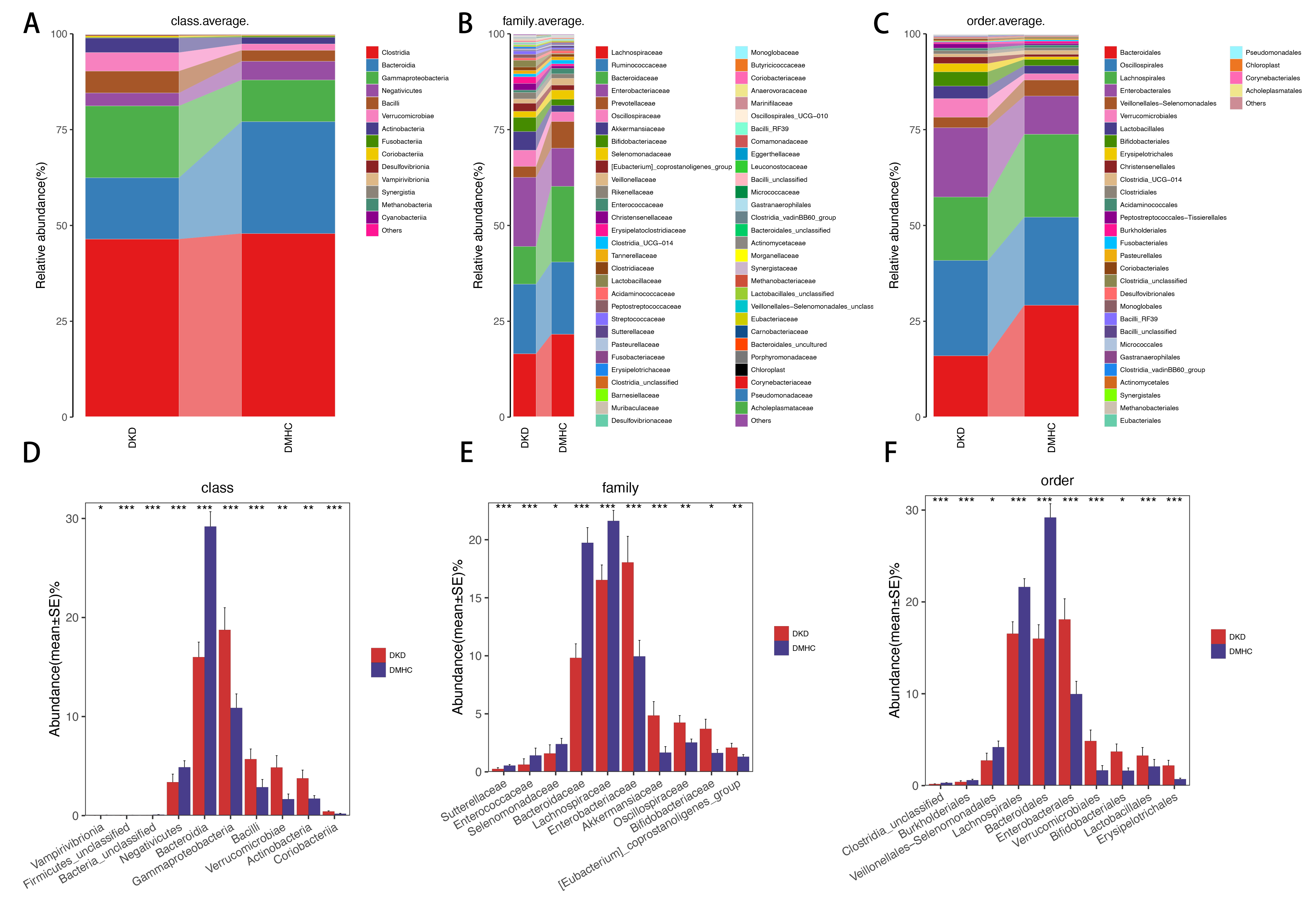

Supplement: Supplementary Figure 3 — Average microbial composition between DKD and non-DKD group were shown at class (A), family (B) or order (C) levels. Microbial comparison among four groups at the class (D), family (E) or order (F) level through Kruskal-Wallis test. [file Image_3.tif]

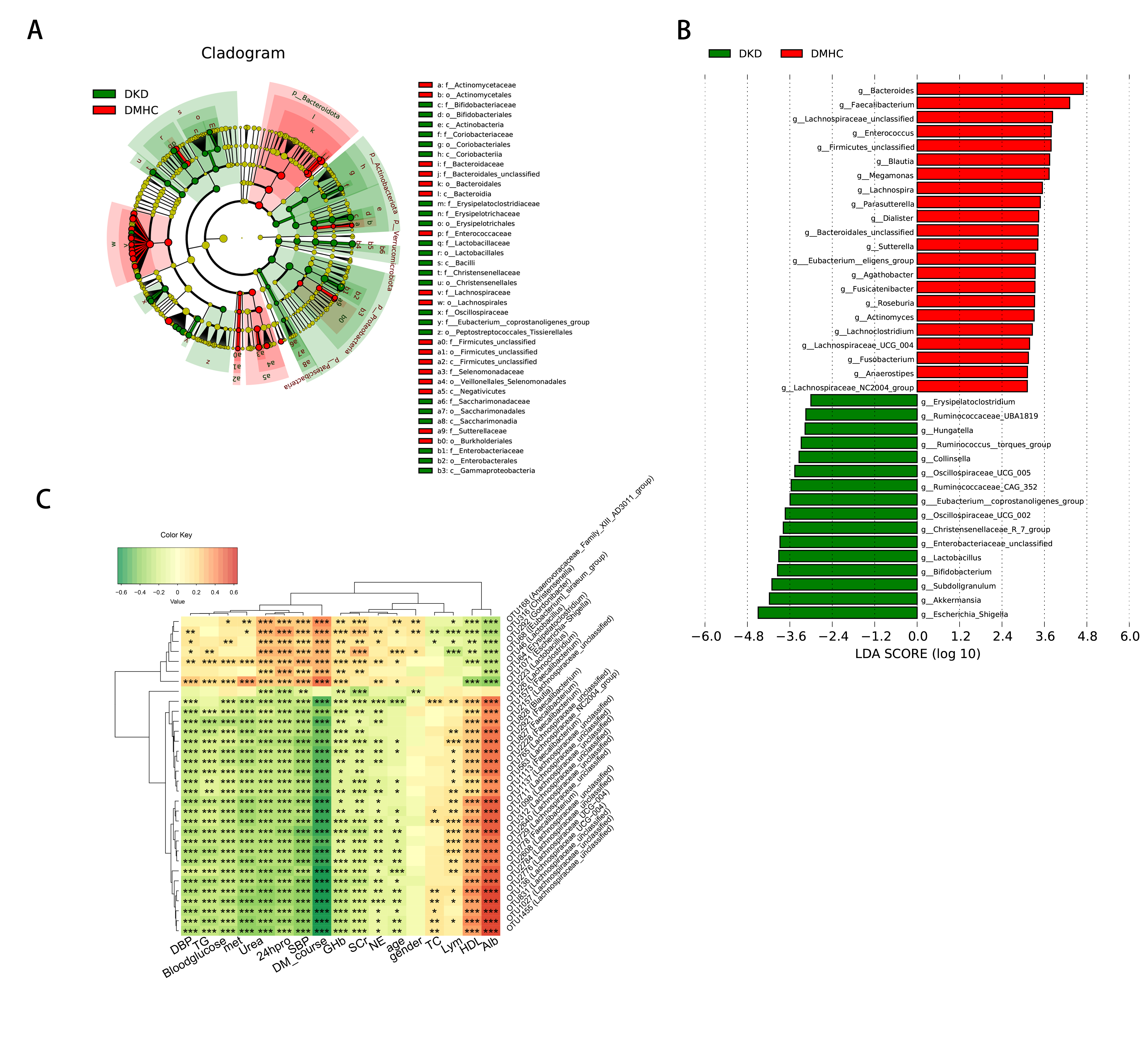

Supplement: Supplementary Figure 4 — Phylogenetic composition and comparison from phylum to genus level were shown in pattern of cladogram (A). LEfSe comparison integrated with bacteria on genus level between DN and MN (B). The length of histogram represented variable importance. LDA score > 2.0, P < 0.05. Spearman’s correlation analysis between crucial clinical parameter and key OTUs when compared DKD patients with non-DKD populations (C). LEfSe, linear discriminate analysis and effect size. [file Image_4.tif]

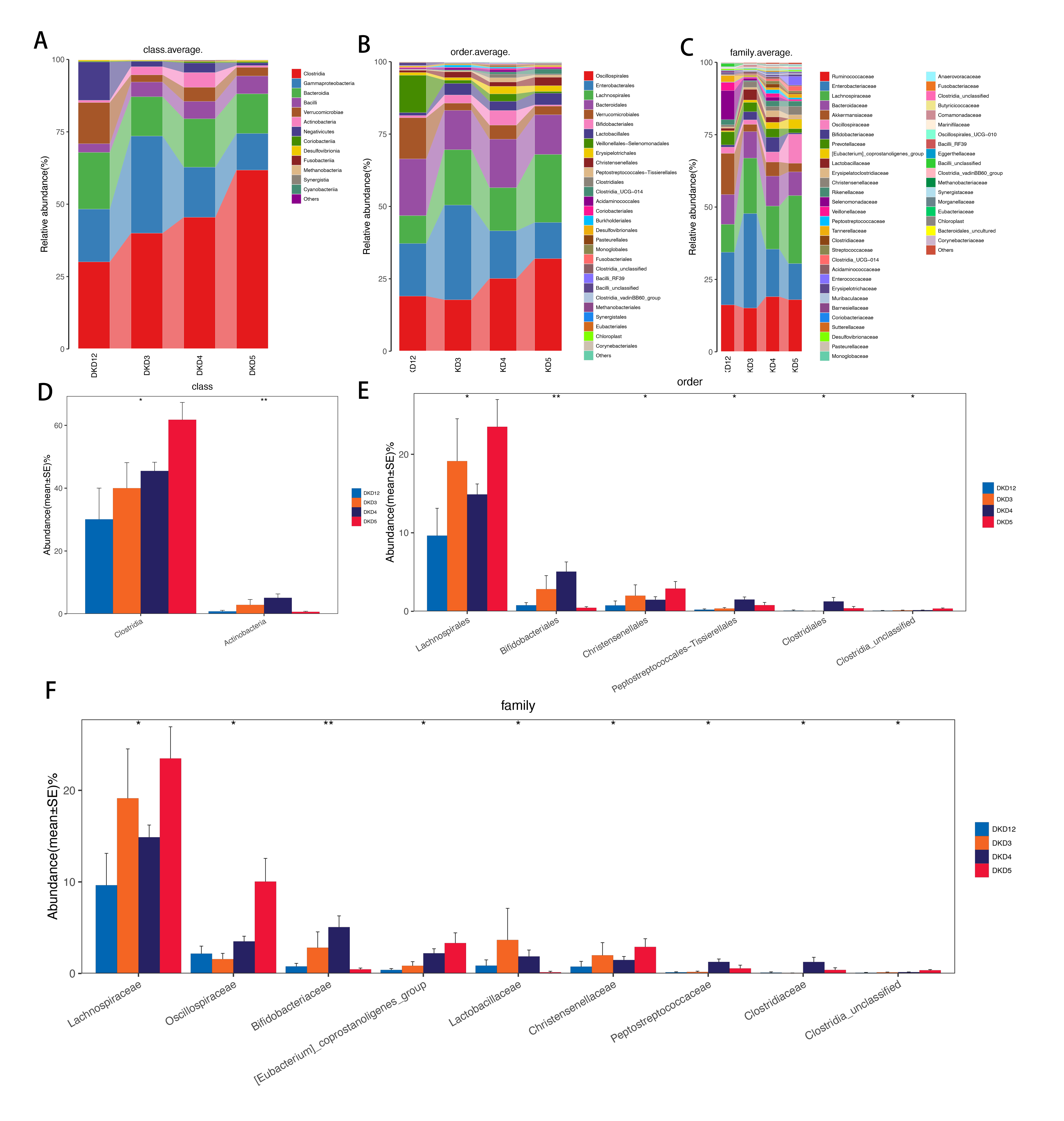

Supplement: Supplementary Figure 5 — Average microbial composition among the DKD1&2, DKD3, DKD4 and DKD5 group were shown class (A), order (B) or family (C) level. Microbial comparison among four groups at the class (D), order (E) or family (F) level through Kruskal-Wallis test. [file Image_5.tif]
