# Supplementary material for: The harmful intestinal microbial community accumulates during DKD exacerbation and microbiome–metabolome combined validation in a mouse model
Source: Front Endocrinol (Lausanne). 2022 Dec 19;13:964389. doi: 10.3389/fendo.2022.964389 (PMC9806430; doi:10.3389/fendo.2022.964389)
Supplement: Supplementary Table 1 — Comparison of α-diversity in discovery cohort (DKD=120 and DMHC=232) [file DataSheet_2.zip › Supplementary tables/Table S12 (POD in discovery corhort).pdf]

	DMHC	DKD
DM10	0.571031	0.428969
DM100	0.435829	0.564171
DM102	0.369628	0.630372
DM103	0.847701	0.152299
DM106	0.504043	0.495957
DM107	0.90107	0.09893
DM108	0.92011	0.07989
DM11	0.76776	0.23224
DM111	0.696477	0.303523
DM112	0.5	0.5
DM113	0.802198	0.197802
DM114	0.083102	0.916898
DM115	0.587079	0.412921
DM116	0.514589	0.485411
DM117	0.506849	0.493151
DM118	0.612137	0.387863
DM119	0.580175	0.419825
DM120	0.960422	0.039578
DM122	0.713924	0.286076
DM123	0.763587	0.236413
DM128	0.790885	0.209115
DM131	0.865079	0.134921
DM132	0.587079	0.412921
DM135	0.335196	0.664804
DM136	0.638655	0.361345
DM137	0.706704	0.293296
DM138	0.637143	0.362857
DM139	0.392857	0.607143
DM15	0.677665	0.322335
DM2	0.628032	0.371968
DM21	0.798942	0.201058
DM22	0.612536	0.387464
DM23	0.754144	0.245856
DM24	0.856749	0.143251
DM26	0.605263	0.394737
DM29	0.792453	0.207547
DM31	0.355844	0.644156
DM32	0.5	0.5
DM33	0.656676	0.343324
DM34	0.335	0.665
DM35	0.502793	0.497207
DM36	0.650273	0.349727
DM37	0.908587	0.091413
DM39	0.671159	0.328841
DM4	0.469274	0.530726
DM41	0.242236	0.757764
DM45	0.716931	0.283069
DM47	0.956522	0.043478
DM48	0.745665	0.254335
DM49	0.518135	0.481865
DM5	0.988764	0.011236
DM50	0.713918	0.286082
DM53	0.882038	0.117962
DM54	0.448649	0.551351
DM57	0.456091	0.543909

DM59	0.755682	0.244318
DM6	0.400568	0.599432
DM60	0.57423	0.42577
DM61	0.790634	0.209366
DM62	0.243553	0.756447
DM64	0.428205	0.571795
DM65	0.968254	0.031746
DM66	0.796407	0.203593
DM68	0.655367	0.344633
DM69	0.741379	0.258621
DM7	0.179361	0.820639
DM70	0.575843	0.424157
DM71	0.944	0.056
DM72	0.677054	0.322946
DM73	0.89863	0.10137
DM75	0.508108	0.491892
DM76	0.523438	0.476563
DM77	0.989446	0.010554
DM78	0.577713	0.422287
DM79	0.205063	0.794937
DM8	0.715818	0.284182
DM81	0.418733	0.581267
DM82	0.403023	0.596977
DM83	0.652893	0.347107
DM84	0.245283	0.754717
DM85	0.847887	0.152113
DM86	0.844875	0.155125
DM87	0.584211	0.415789
DM88	0.454301	0.545699
DM89	0.825	0.175
DM9	0.428169	0.571831
DM90	0.310606	0.689394
DM93	0.541547	0.458453
DM94	0.48285	0.51715
DM96	0.237197	0.762803
DM97	0.319892	0.680108
DM98	0.64497	0.35503
HC1	0.997375	0.002625
HC10	0.970667	0.029333
HC100	0.855114	0.144886
HC101	0.965616	0.034384
HC103	0.965699	0.034301
HC104	0.96978	0.03022
HC105	0.991892	0.008108
HC106	0.904225	0.095775
HC107	0.60733	0.39267
HC108	0.994695	0.005305
HC109	0.997135	0.002865
HC11	1	0
HC111	0.612536	0.387464
HC112	0.904494	0.095506
HC115	0.940379	0.059621
HC116	1	0
HC118	0.739377	0.260623
HC119	0.984211	0.015789
HC120	1	0

HC123	0.981723	0.018277
HC124	0.638356	0.361644
HC125	1	0
HC127	0.778667	0.221333
HC128	0.798928	0.201072
HC13	0.99705	0.00295
HC130	0.85	0.15
HC131	0.986339	0.013661
HC132	1	0
HC133	0.924324	0.075676
HC134	0.997297	0.002703
HC135	0.972067	0.027933
HC137	1	0
HC138	0.99435	0.00565
HC139	1	0
HC14	0.724234	0.275766
HC140	0.041322	0.958678
HC141	0.99734	0.00266
HC142	0.907738	0.092262
HC143	0.742775	0.257225
HC144	0.951289	0.048711
HC145	1	0
HC146	1	0
HC148	0.831884	0.168116
HC15	0.975069	0.024931
HC150	1	0
HC151	0.798928	0.201072
HC153	0.793566	0.206434
HC155	0.541311	0.458689
HC156	0.717808	0.282192
HC157	0.97541	0.02459
HC158	0.906005	0.093995
HC160	0.997151	0.002849
HC162	0.99726	0.00274
HC163	0.879781	0.120219
HC164	0.992308	0.007692
HC165	0.570637	0.429363
HC168	1	0
HC169	0.997207	0.002793
HC17	0.920821	0.079179
HC170	1	0
HC172	0.986877	0.013123
HC173	0.939241	0.060759
HC174	1	0
HC175	0.701449	0.298551
HC178	0.843501	0.156499
HC179	0.88169	0.11831
HC18	1	0
HC183	0.890756	0.109244
HC185	0.942466	0.057534
HC188	1	0
HC189	0.994429	0.005571
HC19	0.991279	0.008721
HC191	0.943343	0.056657
HC192	0.606145	0.393855
HC193	0.708571	0.291429

HC194	0.935233	0.064767
HC196	0.971649	0.028351
HC199	0.977591	0.022409
HC2	0.989474	0.010526
HC200	1	0
HC201	0.976501	0.023499
HC205	0.997389	0.002611
HC206	0.989556	0.010444
HC207	1	0
HC209	1	0
HC21	0.98892	0.01108
HC210	0.951872	0.048128
HC212	0.518414	0.481586
HC24	0.994723	0.005277
HC25	0.887978	0.112022
HC26	0.991736	0.008264
HC27	0.924479	0.075521
HC29	0.988473	0.011527
HC30	1	0
HC31	1	0
HC32	0.997382	0.002618
HC33	1	0
HC35	1	0
HC38	0.988636	0.011364
HC4	0.825301	0.174699
HC40	1	0
HC41	0.997361	0.002639
HC44	0.921622	0.078378
HC45	0.997199	0.002801
HC46	0.873239	0.126761
HC47	0.997297	0.002703
HC49	0.808511	0.191489
HC5	0.913043	0.086957
HC50	1	0
HC51	0.956164	0.043836
HC52	0.7979	0.2021
HC53	0.941667	0.058333
HC54	0.957983	0.042017
HC56	0.994318	0.005682
HC57	1	0
HC59	0.791086	0.208914
HC6	0.865229	0.134771
HC63	0.994429	0.005571
HC64	1	0
HC65	0.931122	0.068878
HC66	1	0
HC69	0.644444	0.355556
HC73	0.972222	0.027778
HC74	1	0
HC76	0.994778	0.005222
HC77	1	0
HC8	0.802168	0.197832
HC80	0.968338	0.031662
HC81	1	0
HC82	0.928	0.072
HC85	1	0

HC86	0.997347	0.002653
HC88	1	0
HC9	0.986413	0.013587
HC90	1	0
HC93	0.986301	0.013699
HC94	0.904762	0.095238
HC95	0.844193	0.155807
HC96	0.994186	0.005814
HC97	1	0
DKD10	0.309392	0.690608
DKD100	0.444737	0.555263
DKD101	0.285319	0.714681
DKD106	0.138667	0.861333
DKD107	0.788462	0.211538
DKD115	0.614243	0.385757
DKD117	0.511335	0.488665
DKD118	0.117493	0.882507
DKD119	0.880556	0.119444
DKD12	0.097257	0.902743
DKD120	0.785908	0.214092
DKD122	0.12963	0.87037
DKD124	0.178947	0.821053
DKD127	0.707736	0.292264
DKD128	0.734637	0.265363
DKD129	0.497368	0.502632
DKD130	0.980392	0.019608
DKD131	0.308094	0.691906
DKD133	0.671958	0.328042
DKD136	0.830239	0.169761
DKD137	0.801587	0.198413
DKD139	0.60221	0.39779
DKD14	0.205405	0.794595
DKD140	0.434659	0.565341
DKD141	0.184697	0.815303
DKD142	0.613445	0.386555
DKD143	0.119481	0.880519
DKD146	0.919689	0.080311
DKD148	0.363636	0.636364
DKD149	0.248082	0.751918
DKD15	0.014663	0.985337
DKD150	0.518617	0.481383
DKD151	0.57672	0.42328
DKD152	0.787402	0.212598
DKD155	0.359551	0.640449
DKD159	0.428962	0.571038
DKD16	0.084469	0.915531
DKD161	0.019553	0.980447
DKD162	0.529891	0.470109
DKD164	0.170029	0.829971
DKD165	0.17847	0.82153
DKD166	0.191304	0.808696
DKD167	0.892265	0.107735
DKD168	0.119593	0.880407
DKD169	0.159383	0.840617
DKD170	0.114973	0.885027
DKD172	0.377778	0.622222

DKD173	0.378917	0.621083
DKD174	0.271003	0.728997
DKD175	0.735537	0.264463
DKD176	0.322034	0.677966
DKD178	0.685294	0.314706
DKD179	0.911111	0.088889
DKD18	0.371429	0.628571
DKD180	0.465714	0.534286
DKD181	0.115068	0.884932
DKD19	0.876344	0.123656
DKD2	0.407008	0.592992
DKD20	0.955959	0.044041
DKD21	0.416446	0.583554
DKD22	0.448468	0.551532
DKD23	0.45614	0.54386
DKD24	0.475578	0.524422
DKD25	0.419689	0.580311
DKD28	0.279891	0.720109
DKD29	0.412088	0.587912
DKD3	0.08871	0.91129
DKD30	0.437838	0.562162
DKD31	0.217848	0.782152
DKD32	0.127778	0.872222
DKD33	0.659517	0.340483
DKD35	0.065903	0.934097
DKD36	0.431217	0.568783
DKD37	0.200528	0.799472
DKD38	0.104439	0.895561
DKD39	0.657216	0.342784
DKD4	0.541667	0.458333
DKD40	0.554974	0.445026
DKD41	0.498615	0.501385
DKD43	0.221024	0.778976
DKD47	0.460674	0.539326
DKD48	0.444444	0.555556
DKD5	0.121447	0.878553
DKD50	0.894587	0.105413
DKD51	0.722955	0.277045
DKD52	0.068493	0.931507
DKD53	0.981481	0.018519
DKD54	0.558989	0.441011
DKD55	0.412533	0.587467
DKD56	0.408719	0.591281
DKD58	0.431319	0.568681
DKD59	0.808219	0.191781
DKD60	0.662234	0.337766
DKD61	0.42663	0.57337
DKD64	0.328947	0.671053
DKD65	0.939828	0.060172
DKD66	0.473538	0.526462
DKD67	0.03876	0.96124
DKD69	0.813187	0.186813
DKD7	0.875	0.125
DKD70	0.173171	0.826829
DKD73	0.116531	0.883469
DKD74	0.425474	0.574526

DKD75	0.563003	0.436997
DKD76	0.162437	0.837563
DKD77	0.061828	0.938172
DKD78	0.726225	0.273775
DKD79	0.191436	0.808564
DKD83	0.430108	0.569892
DKD85	0.026738	0.973262
DKD86	0.472464	0.527536
DKD87	0.864943	0.135057
DKD88	0.148438	0.851563
DKD89	0.382353	0.617647
DKD9	0.639798	0.360202
DKD90	0.161644	0.838356
DKD92	0.298153	0.701847
DKD93	0.169811	0.830189
DKD97	0.083558	0.916442
DKD98	0.187335	0.812665
