# Supplementary material for: The harmful intestinal microbial community accumulates during DKD exacerbation and microbiome–metabolome combined validation in a mouse model
Source: Front Endocrinol (Lausanne). 2022 Dec 19;13:964389. doi: 10.3389/fendo.2022.964389 (PMC9806430; doi:10.3389/fendo.2022.964389)
Supplement: Supplementary Table 1 — Comparison of α-diversity in discovery cohort (DKD=120 and DMHC=232) [file DataSheet_2.zip › Supplementary tables/Table S4 (Phylum).pdf]

ID	DKD.medi	DKD.mea	DKD.se	DMHC.me	DMHC.me	DMHC.se	p-value	z-score
Firmicutes	0.6046(0.37	0.5553534	0.0240208	0.5683(0.38	0.5568328	0.0162484	0.8767993	-0.155028
Bacteroido	0.1085(0.02	0.1600248	0.0151514	0.257(0.076	0.2918835	0.0149905	1.05E-07	-5.318341
Proteobact	0.0673(0.01	0.1876248	0.0223748	0.0137(0.00	0.1090086	0.0141392	1.54E-06	-4.805716
Verrucomi	<0.0001(<(	0.0486002	0.0119398	<0.0001(<(	0.0166261	0.0051715	0.0012992	-3.216149
Actinobact	0.01(0.0035	0.0417901	0.0083142	0.0042(0.00	0.0190468	0.003066	2.98E-05	-4.175306
Fusobacter	<0.0001(<(	0.0018728	0.0012822	<0.0001(<(	0.003297	0.0013789	0.1071045	-1.611345
Desulfoba	0.0005(<0.(	0.0025327	4.41E-04	0.0005(<0.(	0.0016862	2.30E-04	0.4381545	-0.775313
Bacteria_u	0.0002(<0.(	3.92E-04	6.33E-05	0.0005(0.00	9.32E-04	7.50E-05	1.69E-08	-5.641359
Cyanobact	<0.0001(<(	4.60E-04	2.34E-04	<0.0001(<(	4.25E-04	1.48E-04	0.0285412	-2.189766
Synergistor	<0.0001(<(	5.06E-04	1.58E-04	<0.0001(<(	1.61E-04	7.27E-05	5.09E-06	-4.560912
Euryarcha	<0.0001(<(	5.77E-04	2.81E-04	<0.0001(<(	1.70E-05	7.70E-06	4.61E-05	-4.074656
Patescibac	0.0001(<0.(	2.66E-04	4.37E-05	<0.0001(<(	8.16E-05	1.55E-05	2.44E-13	-7.322155
Elusimicro	<0.0001(<(	9.17E-07	9.17E-07	<0.0001(<(	1.93E-06	1.93E-06	0.6406199	-0.466832

Sig_mark	q-value	fixp	fixps
	0.8767993	0.8768	0.8768
***	4.54E-07	<0.0001	***<0.0001
***	5.01E-06	<0.0001	***<0.0001
**	0.0021113	0.0013	** 0.0013
***	6.45E-05	<0.0001	***<0.0001
	0.1392359	0.1071	0.1071
	0.5178189	0.4382	0.4382
***	1.10E-07	<0.0001	***<0.0001
*	0.0412262	0.0285	* 0.0285
***	1.32E-05	<0.0001	***<0.0001
***	8.56E-05	<0.0001	***<0.0001
***	3.17E-12	<0.0001	***<0.0001
	0.6940049	0.6406	0.6406
