# Supplementary material for: The harmful intestinal microbial community accumulates during DKD exacerbation and microbiome–metabolome combined validation in a mouse model
Source: Front Endocrinol (Lausanne). 2022 Dec 19;13:964389. doi: 10.3389/fendo.2022.964389 (PMC9806430; doi:10.3389/fendo.2022.964389)
Supplement: Supplementary Table 1 — Comparison of α-diversity in discovery cohort (DKD=120 and DMHC=232) [file DataSheet_2.zip › Supplementary tables/Table S2 (Beta-diversity).pdf]

Groups	Df	SumsOfSqs	MeanSqs	F.Model	R2	Pr(>F)	Sig_mark
DKD-DMI	1	1.7511502	1.7511502	8.8760428	0.0247329	1.00E-04	***
